# Predictors of the utility of clinical exome sequencing as a first-tier genetic test in patients with Mendelian phenotypes: results from a referral center study on 603 consecutive cases

**DOI:** 10.1186/s40246-023-00455-x

**Published:** 2023-02-05

**Authors:** Tom Alix, Céline Chéry, Thomas Josse, Jean-Pierre Bronowicki, François Feillet, Rosa-Maria Guéant-Rodriguez, Farès Namour, Jean-Louis Guéant, Abderrahim Oussalah

**Affiliations:** 1 Division of Biochemistry, Molecular Biology, and Nutrition, Department of Molecular Medicine, University Hospital of Nancy, 54000 Nancy, France; 2 INSERM UMR_S 1256, Nutrition, Genetics, and Environmental Risk Exposure (NGERE), Faculty of Medicine of Nancy, University of Lorraine, 9 Avenue de la Forêt de Haye, 54000 Nancy, France; 3 Reference Center for Inborn Errors of Metabolism (ORPHA67872), University Hospital of Nancy, 54000 Nancy, France; 4 Department of Gastroenterology and Liver Diseases, University Hospital of Nancy, 54000 Nancy, France; 5 Department of Pediatrics, University Hospital of Nancy, 54000 Nancy, France

**Keywords:** Clinical exome sequencing, Diagnostic yield, Predictors of clinical utility, Mendelian phenotype, Reference center, Consecutive case series

## Abstract

**Background:**

Clinical exome sequencing (CES) provides a comprehensive and effective analysis of relevant disease-associated genes in a cost-effective manner compared to whole exome sequencing. Although several studies have focused on the diagnostic yield of CES, no study has assessed predictors of CES utility among patients with various Mendelian phenotypes. We assessed the effectiveness of CES as a first-level genetic test for molecular diagnosis in patients with a Mendelian phenotype and explored independent predictors of the clinical utility of CES.

**Results:**

Between January 2016 and December 2019, 603 patients (426 probands and 177 siblings) underwent CES at the Department of Molecular Medicine of the University Hospital of Nancy. The median age of the probands was 34 years (IQR, 12–48), and the proportion of males was 46.9% (200/426). Adults and children represented 64.8% (276/426) and 35.2% (150/426), respectively. The median test-to-report time was 5.6 months (IQR, 4.1–7.2). CES revealed 203 pathogenic or likely pathogenic variants in 160 patients, corresponding to a diagnostic yield of 37.6% (160/426). Independent predictors of CES utility were criteria strongly suggestive of an extreme phenotype, including pediatric presentation and patient phenotypes associated with an increased risk of a priori probability of a monogenic disorder, the inclusion of at least one family member in addition to the proband, and a CES prescription performed by an expert in the field of rare genetic disorders.

**Conclusions:**

Based on a large dataset of consecutive patients with various Mendelian phenotypes referred for CES as a first-tier genetic test, we report a diagnostic yield of ~ 40% and several independent predictors of CES utility that might improve CES diagnostic efficiency.

**Supplementary Information:**

The online version contains supplementary material available at 10.1186/s40246-023-00455-x.

## Background

Rare diseases affect more than 400 million people worldwide, corresponding to an estimated cumulative population prevalence of ≈ 3.5 to 5.9%, according to the Orphanet database [1]. Genetic disorders represent 72% of rare diseases, and 70% have an exclusive pediatric onset [1]. The advent of high-throughput sequencing methods for DNA analysis has revolutionized the diagnostic approach for patients with suspected genetic disorders [2]. In recent years, several studies have reported the usefulness of clinical exome sequencing (CES) [2–9], whole-exome sequencing (WES) [10–15], and whole-genome sequencing (WGS) [14–20] in patients with highly suggestive Mendelian phenotypes, reporting diagnostic yields ranging from 9 to 61%, 32 to 74%, and 16 to 42%, respectively. In neonates and infants with critically ill conditions, CES, WES, or WGS approaches have achieved a molecular diagnostic rate of 37 to 72% [21–29]. CES provides a comprehensive and effective analysis of relevant disease-associated genes in a cost-effective manner compared to whole exome sequencing.

Studies to date assessing high-throughput sequencing methods for obtaining molecular diagnoses have focused on the diagnostic rate as the primary outcome, whereas few studies have examined whether baseline patient characteristics are able to predict exome sequencing efficiency for establishing a molecular diagnosis. However, to our knowledge, no study has assessed predictors of the clinical utility of exome sequencing, including the diagnostic yield, confirmation of the suspected clinical diagnosis, and therapeutic guidance based on a molecular diagnosis report.

Here, we report real-life experience involving more than 600 CES analyses performed at a referral center as a first-tier genetic test among adult and pediatric patients with various Mendelian phenotypes, including inherited metabolic disorders. We assessed CES efficiency for achieving a molecular diagnosis as well as independent predictors of CES clinical utility.

## Results

### Study population

Between January 2016 and December 2019, 603 patients (426 probands and 177 siblings) underwent CES at the Department of Molecular Medicine of the University Hospital of Nancy (Table 1). The median age of the probands was 34 years (IQR, 12–48), and the proportion of males was 46.9% (200/426). The majority of CES prescriptions originated from the University Hospital of Nancy (96.7%, 412/426), mainly from outpatient clinics (83.3%, 355/426) and hospital departments (7.5%, 32/426). Adult and pediatric populations represented 64.8% (276/426) and 35.2% (150/426), respectively. Eighty percent of CES analyses were performed for patients followed at the Reference Centre for Inborn Errors of Metabolism (53.8%, 229/426), the Department of Gastrointestinal & Liver Diseases (15.7%, 67/426), or the Department of Endocrinology and Nutrition (13.4%, 57/426) (Table 1). The main suspected diagnoses in relation to CES prescription were metabolic disorders (40.6%, 173/426), dyslipidemia (17.8%, 76.426), liver and biliary tract disorders (15.3%, 65/426), and neurological disorders (6.3%, 27/426) (Table 2). One-carbon metabolism disorders represented 24.4% of CES indications (104/426) and more than 60% of CES performed in the setting of ‘Metabolic disorders’ (104/173). Among patients with dyslipidemia, more than 85% (66/76) of CES analyses were performed for those exhibiting hypercholesterolemia (46/66, 69.7%) or hypertriglyceridemia (20/66, 30.3%) (Table 2). Among patients with liver or biliary tract disorders, CES analyses were mainly performed to explore hyperferritinemia (19/426, 4.5%), suspicion of low-phospholipid-associated cholelithiasis syndrome (12/426, 2.8%), or a cholestatic disorder (12/426, 2.8%), totaling 66% (43/65) of CES indications in this subgroup (Table 2).

**Table 1:** Description of 426 patients assessed using clinical exome sequencing at the Department of Molecular Medicine of the University Hospital of Nancy

Demographics			
Age at clinical exome sequencing (years)—n, median (IQR)	426	34	(12–48)
Female—n/N, % (95% CI)	226/426	53.1	(48.3–57.8)
Male—n/N, % (95% CI)	200/426	46.9	(42.2–51.7)
Institution—n/N, % (95% CI)			
University hospital	412/426	96.7	(95.0–98.4)
Regional hospital	13/426	3.1	(1.4–4.7)
Private practice	1/426	0.2	(0*–0.7)
Setting—n/N, % (95% CI)			
Outpatient clinic, University Hospital of Nancy	355/426	83.3	(79.8–86.9)
Hospital department, University Hospital of Nancy	32/426	7.5	(5.0–10.0)
Medical day hospital, University Hospital of Nancy	7/426	1.7	(0.4–2.9)
Outside the University Hospital of Nancy	32/426	7.5	(5.0–10.0)
Adult, pediatric departments—n/N, % (95% CI)			
Adult care department	276/426	64.8	(60.2–69.3)
Pediatric care department	150/426	35.2	(30.7–39.8)
Geographical region and city—n/N, % (95% CI)			
Nancy	394/426	92.5	(90.0–95.0)
North-East region, outside Nancy	16/426	3.8	(1.9–5.6)
Other	16/426	3.8	(1.9–5.6)
Department—n/N, % (95% CI)			
Reference Centre for Inborn Errors of Metabolism (RCIEM)	229/426	53.8	(49.0–58.5)
Gastrointestinal & Liver diseases	67/426	15.7	(12.3–19.2)
Endocrinology & Nutrition	57/426	13.4	(10.1–16.6)
Pediatrics (outside, RCIEM)	20/426	4.7	(2.7–6.7)
Neurology	12/426	2.8	(1.2–4.4)
Internal Medicine	11/426	2.6	(1.1–4.1)
Clinical Genetics	8/426	1.9	(0.6–3.2)
Vascular Medicine	5/426	1.2	(0.1–2.2)
Cardiology	4/426	0.9	(0–1.9)
Hematology	4/426	0.9	(0–1.9)
Nephrology	3/426	0.7	(0*–1.5)
Hematology-oncology	2/426	0.5	(0*–1.1)
Geriatrics	1/426	0.2	(0*–0.7)
Gynecology and Obstetrics	1/426	0.2	(0*–0.7)
Orthopedics	1/426	0.2	(0*–0.7)
Intensive Care	1/426	0.2	(0*–0.7)

*IQR* interquartile range; *95% CI* 95% confidence interval

**Table 2:** Description of suspected diagnoses associated with clinical exome sequencing prescriptions

Suspected diagnoses			
Metabolic disorders—n/N, % (95% CI)	173/426	40.6	(35.9–45.3)
One-carbon metabolism disorders	104/426	24.4	(20.3–28.5)
Energy metabolism disorders	9/426	2.1	(0.7–3.5)
Organic acidurias	6/426	1.4	(0.3–2.5)
Congenital hyperinsulinisms	6/426	1.4	(0.3–2.5)
Lysosomal storage disorders*	6/426	1.4	(0.3–2.5)
Glycogen storage diseases	5/426	1.2	(0.1–2.2)
Hyperbilirubinemias	5/426	1.2	(0.1–2.2)
Peroxisomal disorders	5/426	1.2	(0.1–2.2)
Biopterin metabolism disorders	5/426	1.2	(0.1–2.2)
Calcium and phosphorus metabolic disorders	5/426	1.2	(0.1–2.2)
Alkaptonuria	3/426	0.7	(0†–1.5)
Metabolic disorders, miscellaneous	14/426	3.3	(1.6–5.0)
Dyslipidemia	76/426	17.8	(14.2–21.5)
Hypercholesterolemia	46/426	10.8	(7.8–13.8)
Hypertriglyceridemia	20/426	4.7	(2.7–6.7)
Hypolipoproteinemia	5/426	1.2	(0.1–2.2)
Mixed dyslipidemia	2/426	0.5	(0†–1.1)
Dyslipidemia, Other	3/426	0.7	(0†–1.5)
Liver and biliary tract disorders	65/426	15.3	(12.0–19.1)
Hyperferritinemia	19/426	4.5	(2.5–6.4)
Low phospholipid-associated cholelithiasis	12/426	2.8	(1.2–4.4)
Cholestatic disorders	12/426	2.8	(1.2–4.4)
Wilson’s disease	9/426	2.1	(0.7–3.5)
Cryptogenic cirrhosis	4/426	0.9	(0–1.9)
Chronic liver cytolysis	4/426	0.9	(0–1.9)
Alpha-1-antitrypsin deficiency	2/426	0.5	(0†–1.1)
Polycystic liver disease	2/426	0.5	(0†–1.1)
Liver steatosis	1/426	0.2	(0†–0.7)
Neurological disorder	27/426	6.3	(4.0–8.7)
Ataxia, hypotonia, paraparesis	13/426	3.1	(1.4–4.7)
Mental retardation with or without autism	5/426	1.2	(0.1–2.2)
Epilepsy	3/426	0.7	(0†–1.5)
Neurological disorders, other	6/426	1.4	(0.3–2.5)
Inflammatory and autoinflammatory disease	18/426	4.2	(2.3–6.1)
Autoinflammatory diseases	17/426	4.0	(2.1–5.9)
Inflammatory diseases, other	1/426	0.2	(0†–0.7)
Developmental abnormality	13/426	3.1	(1.4–4.7)
Heart defects	4/426	0.9	(0–1.9)
Neural tube defects	3/426	0.7	(0†–1.5)
Developmental abnormality, other	6/426	1.4	(0.3–2.5)
Mitochondrial cytopathy	8/426	1.9	(0.6–3.2)
Pancreatitis	8/426	1.9	(0.6–3.2)
Intestinal absorption disorders	7/426	1.6	(0.4–2.9)
Myopathy	7/426	1.6	(0.4–2.9)
Osteogenesis imperfecta	7/426	1.6	(0.4–2.9)
Lipodystrophy	5/426	1.2	(0.1–2.2)
Primary immunodeficiencies	4/426	0.9	(0–2.2)
Other	8/426	1.9	(0.6–3.2)
Thrombophilia	2/426	0.5	(0†–1.1)
Amyloidosis	1/426	0.2	(0†–0.7)
Cancer	2/426	0.5	(0†–1.1)
Marfan syndrome	1/426	0.2	(0†–0.7)
Sudden death	1/426	0.2	(0†–0.7)
Telomere Diseases	1/426	0.2	(0†–0.7)

*n* number of observations; *N* total number of patients; *95% CI* 95% confidence interval

^*^Lysosomal storage disorders other than glycogen storage disease type II

^†^The 95% CI was truncated at the left margin

### Diagnostic yield of clinical exome sequencing

**Fig. 1:**
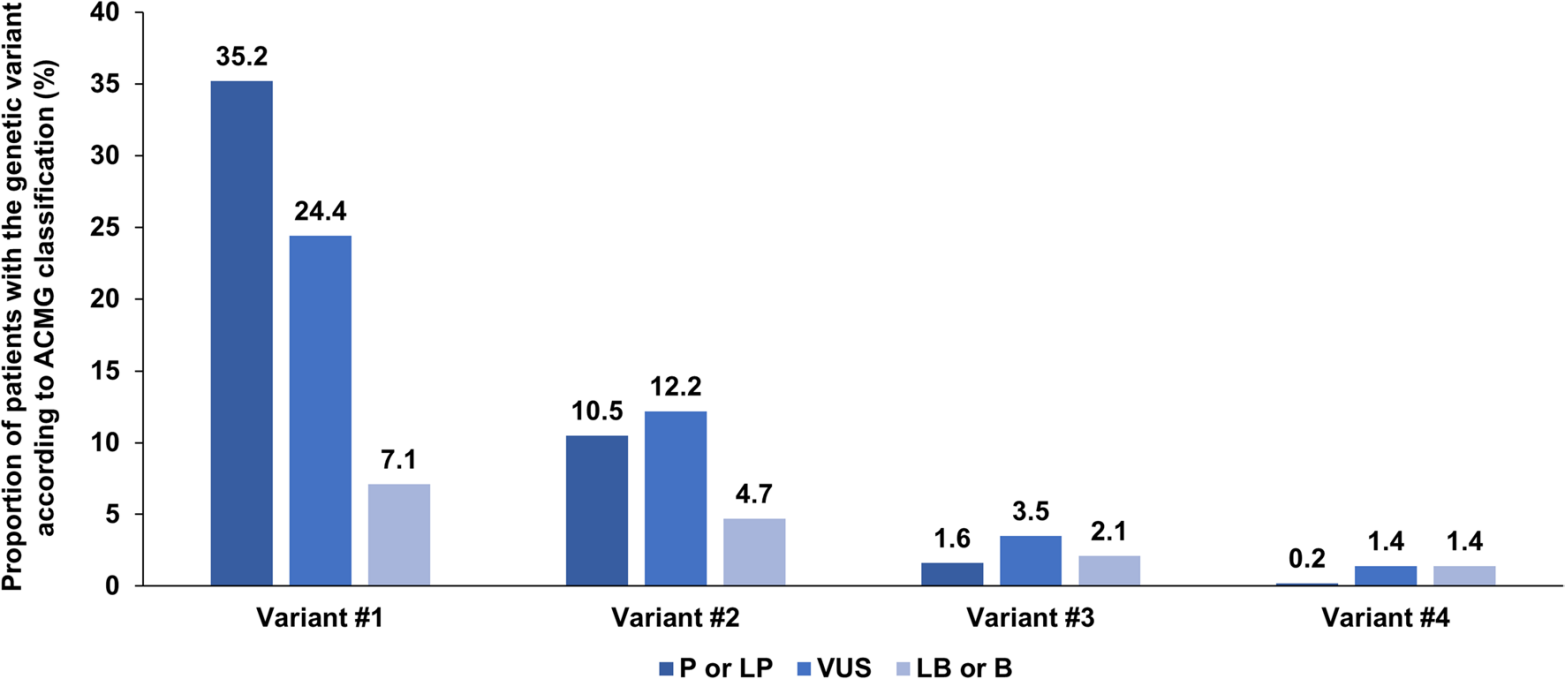
Distribution of genetic variants according to their pathogenicity. Variants #1 to #4 are ordered as in the CES medical report

**Fig. 2:**
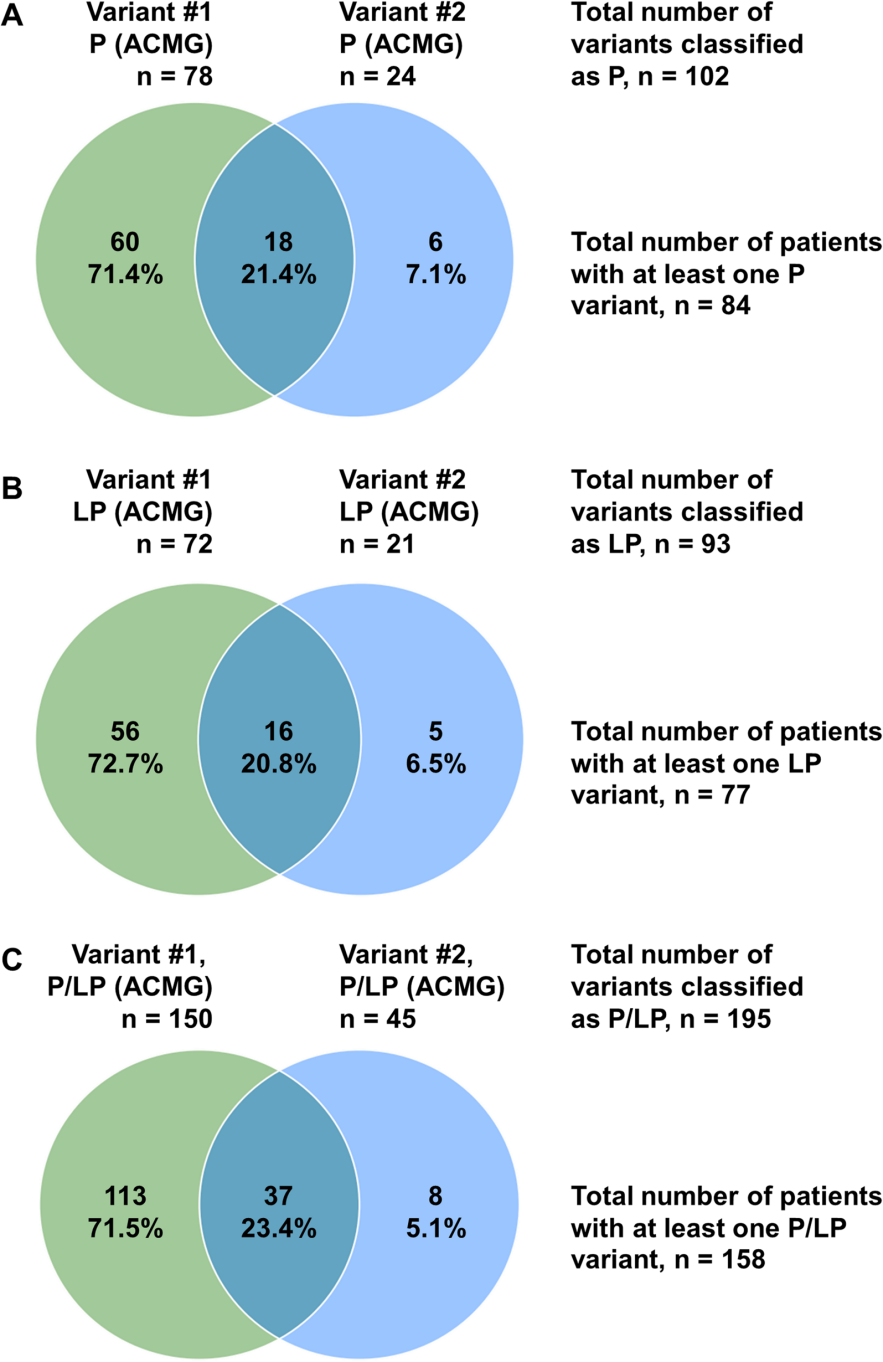
Distribution of the 195 pathogenic or likely pathogenic (P/LP) variants among 158 patients exhibiting at least one P/LP variant (variants #1 and #2 correspond to the first two variants in the CES medical report). The numbers outside the Venn diagram correspond to the numbers of genetic variants. The numbers within the Venn diagram correspond to the number of patients. Panels **A**–**C** show numbers for P, LP, and P/LP variants

The median test-to-report time was 5.6 months (IQR, 4.1–7.2), and the median number of genetic variants reported per patient in the CES was 1 (IQR, 0–2); the data were 1 (IQR, 0–1) and 0 (IQR, 0–1) when P/LP/VUS variants or only P/LP variants were considered, respectively. The proportions of patients with genetic variants according to the ACMG classification are reported in Table 3, Fig. 1, and Additional file 1: Figure S1. CES revealed 203 P/LP variants in 160 patients, corresponding to a diagnostic yield of 37.6% (160/426) (Table 3 and Additional file 1: Figure S2), and this figure increased to 60.8% (259/426) when VUSs were included in diagnostic yield analysis (Table 3). The first two variants (variants #1 and #2) classified as P/LP represented 96.1% (195/203) of all P/LP variants reported (Fig. 2 and Additional file 1: Figure S2).

**Table 3:** Results of clinical exome sequencing clinical reports for 426 assessed patients

Time delay between CES and report (Months)—n, median (IQR)	419	5.6	(4.1–7.2)
Description of genetic variants in the clinical exome sequencing report			
Proportion of patients with at last one variant classified as LP or P—n/N, % (95% CI)	160/426	37.6%	(32.9–42.2)
Proportion of patients with at last one variant classified as VUS, LP, or P—n/N, % (95% CI)	259/426	60.8%	(56.1–65.5)
Number of genetic variants reported in the CES report per patient—n, median (IQR)	426	1	(0–2)
Number of genetic variants classified as VUS, LP, or P per patient—n, median (IQR)	426	1	(0–1)
Number of genetic variants classified as LP or P per patient—n, median (IQR)	426	0	(0–1)
Variant #1			
ACMG, pathogenic—n/N, % (95% CI)	78/426	18.3%	(14.6–22)
ACMG, likely pathogenic—n/N, % (95% CI)	72/426	16.9%	(13.3–20.5)
ACMG, uncertain significance—n/N, % (95% CI)	104/426	24.4%	(20.3–28.5)
ACMG, likely benign—n/N, % (95% CI)	8/426	1.9%	(0.6–3.2)
ACMG, benign—n/N, % (95% CI)	22/426	5.2%	(3.1–7.3)
No variant retrieved—n/N, % (95% CI)	142/426	33.2%	(28.8–37.8)
NGS-diag network scoring system—n, median (IQR)	284	4	(3–5)
Variant #2			
ACMG, pathogenic—n/N, % (95% CI)	24/426	5.6%	(3.4–7.8)
ACMG, likely pathogenic—n/N, % (95% CI)	21/426	4.9%	(2.9–7.0)
ACMG, uncertain significance—n/N, % (95% CI)	52/426	12.2%	(9.1–15.3)
ACMG, likely benign—n/N, % (95% CI)	3/426	0.7%	(0–1.5)
ACMG, benign—n/N, % (95% CI)	17/426	4.0%	(2.1–5.9)
No variant retrieved—n/N, % (95% CI)	309/426	72.5%	(68.3–76.8)
NGS-diag network scoring system—n, median (IQR)	117	3	(3–4)
Variant #3			
ACMG, pathogenic—n/N, % (95% CI)	1/426	0.2%	(0–0.6)
ACMG, likely pathogenic—n/N, % (95% CI)	6/426	1.4%	(0.3–2.5)
ACMG, uncertain significance—n/N, % (95% CI)	15/426	3.5%	(1.8–5.3)
ACMG, likely benign—n/N, % (95% CI)	1/426	0.2%	(0–0.7)
ACMG, benign—n/N, % (95% CI)	8/426	1.9%	(0.6–3.2)
No variant retrieved—n/N, % (95% CI)	395/426	92.7%	(89.8–95.0)
NGS-diag network scoring system—n, median (IQR)	31	3	(1–3)
Variant #4			
ACMG, pathogenic—n/N, % (95% CI)	0/426	0%	(—)
ACMG, likely pathogenic—n/N, % (95% CI)	1/426	0.2%	(0–0.6)
ACMG, uncertain significance—n/N, % (95% CI)	6/426	1.4%	(0.3–2.5)
ACMG, likely benign—n/N, % (95% CI)	1/426	0.2%	(0–0.6)
ACMG, benign—n/N, % (95% CI)	5/426	1.2%	(0.1–2.2)
No variant retrieved—n/N, % (95% CI)	413/426	96.9%	(94.7–98.3)
NGS-diag network scoring system—n, median (IQR)	13	3	(1–3)
Confirmation of the clinically suspected diagnosis—n/N, % (95% CI)			
Confirmation of the suspected diagnosis	183/426	43.0%	(38.2–47.7)
Contribution to the suspected diagnosis	32/426	7.5%	(5.0–10.0)
No contributive variant to the suspected diagnosis	211/426	49.5%	(44.8–54.3)
Therapy guided by NGS results—n/N, % (95% CI)			
Yes	62/426	14.6%	(11.2–17.9)
No	263/426	61.7%	(57.1–66.4)
No follow-up information	101/426	23.7%	(19.7–27.8)

*n* number of observations; *N* total number of patients; *95% CI* 95% confidence interval; *IQR* interquartile range, 25th–75th

**Fig. 3:**
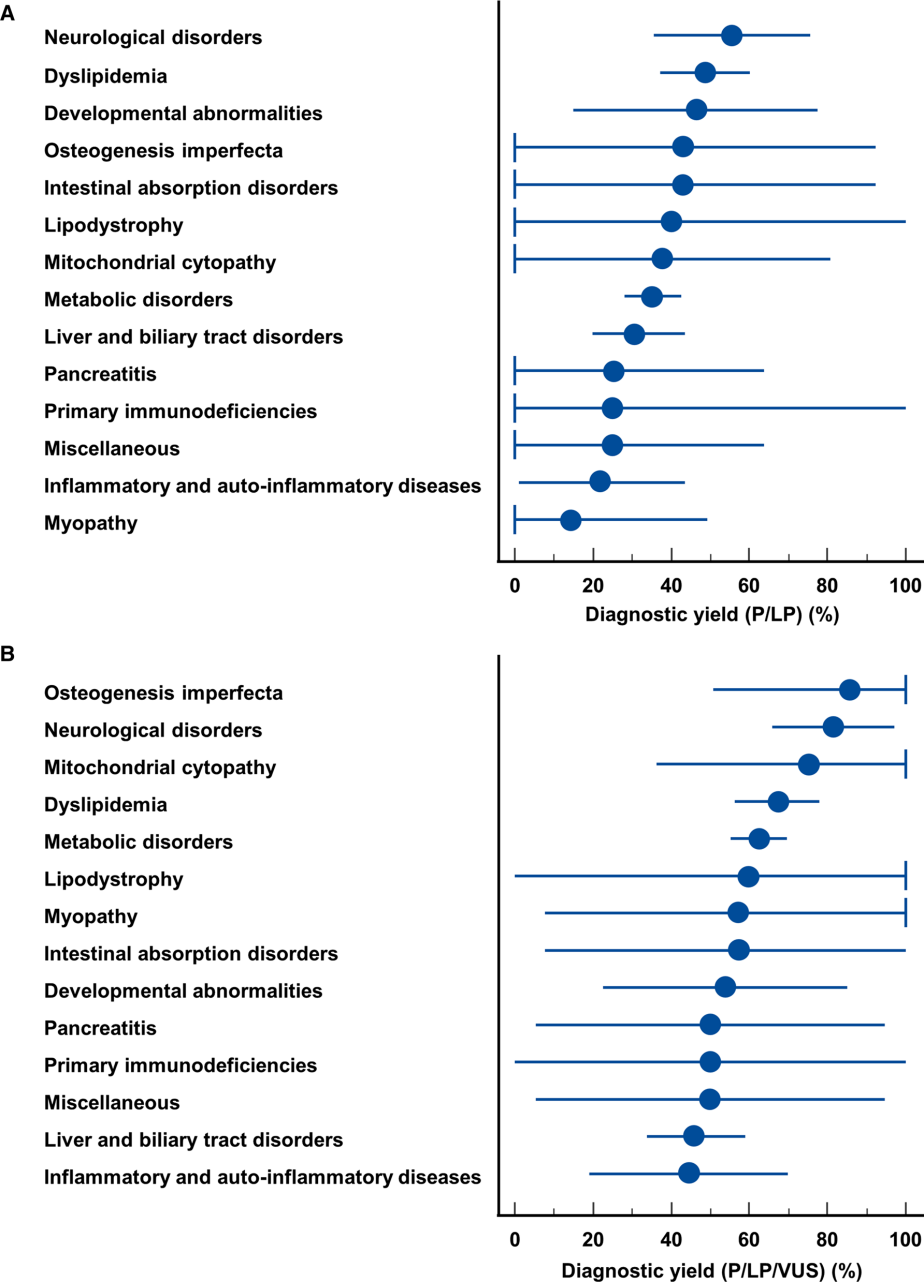
Diagnostic yield of CES according to the suspected diagnosis when only pathogenic or likely pathogenic (P/LP) variants were considered (Panel **A**) or when variants of uncertain significance (VUS) were included in the diagnostic yield analysis (Panel **B**). Vertical bars denote truncated 95% confidence intervals at the right or the left margin

The diagnostic yield of CES varied according to the suspected diagnosis (Table 4). When P/LP variants were considered, the CES diagnostic yield was > 40% for patients with neurological disorders, dyslipidemia, developmental abnormalities, osteogenesis imperfecta, intestinal absorption disorders, or lipodystrophy. When VUSs were included in the evaluation of CES efficiency, all suspected diagnosis subgroups had a diagnostic yield above 40%, with the top items represented by osteogenesis imperfecta, neurological disorders, mitochondrial cytopathy, dyslipidemia, inherited metabolic disorders, and lipodystrophy (Fig. 3).

**Table 4:** diagnostic yield of clinical exome sequencing according to the suspected diagnosis

Metabolic disorders—n/N, % (95% CI)			
At least one variant classified as VUS, LP, or P	108/173	62.4	(55.1–69.7)
At least one variant classified as LP or P	61/173	35.3	(28.1–42.5)
Dyslipidemia—n/N, % (95% CI)			
At least one variant classified as VUS, LP, or P	51/76	67.1	(56.3–77.9)
At least one variant classified as LP or P	37/76	48.7	(37.2–60.2)
Liver and biliary tract disorders—n/N, % (95% CI)			
At least one variant classified as VUS, LP, or P	30/65	46.2	(33.7–59.0)
At least one variant classified as LP or P	20/65	30.8	(19.9–43.5)
Neurological disorders—n/N, % (95% CI)			
At least one variant classified as VUS, LP, or P	22/27	81.5	(65.8–97.1)
At least one variant classified as LP or P	15/27	55.6	(35.5–75.6)
Inflammatory and autoinflammatory diseases—n/N, % (95% CI)			
At least one variant classified as VUS, LP, or P	8/18	44.4	(19.0–69.9)
At least one variant classified as LP or P	4/18	22.2	(0.9–43.5)
Developmental abnormalities—n/N, % (95% CI)			
At least one variant classified as VUS, LP, or P	7/13	53.8	(22.5–85.2)
At least one variant classified as LP or P	6/13	46.2	(14.8–77.5)
Mitochondrial cytopathy—n/N, % (95% CI)			
At least one variant classified as VUS, LP, or P	6/8	75	(36.3–100*)
At least one variant classified as LP or P	3/8	37.5	(0–80.8)
Pancreatitis—n/N, % (95% CI)			
At least one variant classified as VUS, LP, or P	4/8	50	(5.3–94.7)
At least one variant classified as LP or P	2/8	25	(0–63.7)
Myopathy—n/N, % (95% CI)			
At least one variant classified as VUS, LP, or P	4/7	57.1	(7.7–100*)
At least one variant classified as LP or P	1/7	14.3	(0–49.2)
Osteogenesis imperfecta—n/N, % (95% CI)			
At least one variant classified as VUS, LP, or P	6/7	85.7	(50.8–100*)
At least one variant classified as LP or P	3/7	42.9	(0–92.3)
Intestinal absorption disorders—n/N, % (95% CI)			
At least one variant classified as VUS, LP, or P	4/7	57.1	(7.7–100)
At least one variant classified as LP or P	3/7	42.9	(†0–92.3)
Lipodystrophy—n/N, % (95% CI)			
At least one variant classified as VUS, LP, or P	3/5	60	(0–100*)
At least one variant classified as LP or P	2/5	40	(0–100*)
Primary immunodeficiencies—n/N, % (95% CI)			
At least one variant classified as VUS, LP, or P	2/4	50	(0–100)
At least one variant classified as LP or P	1/4	25	(0–100)
Miscellaneous conditions—n/N, % (95% CI)			
At least one variant classified as VUS, LP, or P	4/8	50	(5.3–94.7)
At least one variant classified as LP or P	2/8	25	(0–63.7)

*n* number of observations; *N* total number of patients; *95% CI* 95% confidence interval

*The 95% CI was truncated at the right margin

^†^The 95% CI was truncated at the left margin

### Predictors of CES efficiency in univariate and multivariable analyses

#### Predictors of discovering at least one P/LP variant

In univariate analyses, several items were associated with discovering at least one genetic variant classified as P/LP (Table 5). Among them, three maintained their significance in multivariable analysis: ‘Suspected diagnosis subgroup, Lysosomal disorder’ (OR, 11.81 [95% CI, 1.35–103.33]; *P* = 0.03), ‘Suspected diagnosis subgroup, Hypercholesterolemia’ (OR, 4.14 [95% CI, 2.15–7.96]; *P* < 0.0001), and ‘Pediatric care department’ (OR, 1.96 [95% CI, 1.28–2.99]; *P* = 0.002) (Table 5). The results of univariate and multivariate analyses assessing predictors of retrieving at least one P/LP/VUS variant are reported in Additional file 1: Table S2.

**Table 5:** Predictors of discovering at least one variant classified as likely pathogenic or pathogenic in univariate and multivariable analyses

Predictor	Univariate analysis			Multivariable analysis†		
	Beta (SE)	Odds ratio (95% CI)	*P* value*	Beta (SE)	Odds ratio (95% CI)	*P* value‡
Suspected diagnosis subgroup, Lysosomal disorder	2.15 (1.1)	8.55 (0.99–73.84)	2.01 × 10^−2^	2.47 (1.11)	11.81 (1.35–103.33)	0.03
Suspected diagnosis subgroup, Hypercholesterolemia	1.28 (0.33)	3.61 (1.9–6.86)	5.62 × 10^−5^	1.42 (0.33)	4.14 (2.15–7.96)	< 0.0001
Pediatric care department	0.55 (0.21)	1.73 (1.15–2.6)	8.29 × 10^−3^	0.67 (0.22)	1.96 (1.28–2.99)	0.002
Suspected diagnosis subgroup, cholestatic disorder	− 1.93 (1.05)	0.15 (0.02–1.14)	1.84 × 10^−2^	Not retained§		
Suspected diagnosis group, Dyslipidemia	0.56 (0.26)	1.75 (1.06–2.89)	2.89 × 10^−2^	Not used in the model||		
Adult care department	− 0.55 (0.21)	0.58 (0.38–0.87)	8.29 × 10^−3^	Not used in the model||		

*95% CI* 95% confidence interval; *Beta* beta coefficient; *SE* standard error

^*^Univariate logistic regression analysis

^†^Cox & Snell *R*^2^, 0.07; Nagelkerke *R*^2^, 0.10; Percent of cases correctly classified, 67%; AUROC, 0.629 (95% CI, 0.581 to 0.675)

^‡^ Multivariable logistic regression analysis using the stepwise method

^§^ Not retained in the multivariate logistic regression model

|| Items not used in multivariable logistic regression analysis to avoid collinearity with other items reported in the multivariable model (e.g., ‘Adult care department’ vs. ‘Pediatric care department’)

#### Predictors of confirming the suspected clinical diagnosis

The CES report confirmed the suspected clinical diagnosis in 43.0% (183/426) of the patients and contributed to the diagnosis in 7.5% (32/426) (Table 3). In univariate analyses, several items were associated with a confirmation or a contribution to the suspected clinical diagnosis (Table 6), with three maintaining significance in multivariable analysis: ‘Suspected diagnosis subgroup, Hypercholesterolemia’ (OR, 2.94 [95% CI, 1.52–5.70]; *P* = 0.001), ‘Exome sequencing for the proband and at least one family member’ (OR, 1.90 [95% CI, 1.20–3.01]; *P* = 0.007), and ‘Suspected diagnosis group, Liver disorder’ (OR, 0.30 [95% CI, 0.14–0.65]; *P* = 0.002).

**Table 6:** Predictors of confirming the suspected clinical diagnosis using clinical exome sequencing in univariate and multivariable analyses

	Univariate analysis			Multivariable analysis†		
	Beta (SE)	Odds ratio (95% CI)	*P* value*	Beta (SE)	Odds ratio (95% CI)	*P* value‡
Suspected diagnosis subgroup, Hypercholesterolemia	1.02 (0.34)	2.76 (1.41–5.41)	1.92 × 10^−3^	1.08 (0.34)	2.94 (1.52–5.70)	0.001
Exome sequencing on the proband and at least one family member	0.81 (0.23)	2.25 (1.42–3.56)	4.27 × 10^−4^	0.64 (0.24)	1.90 (1.20–3.01)	0.007
Suspected diagnosis group, Liver disorder	− 1.29 (0.34)	0.28 (0.14–0.53)	3.81 × 10^−5^	− 1.19 (0.39)	0.30 (0.14–0.65)	0.002
Suspected diagnosis group, Osteogenesis imperfecta	1.8 (1.08)	6.03 (0.72–50.51)	4.75 × 10^−2^	Not retained§		
Suspected diagnosis subgroup, One-carbon metabolism disorder	0.65 (0.23)	1.91 (1.21–3.01)	4.59 × 10^−3^	Not retained§		
Department, Reference Center for Inborn Errors of Metabolism	0.59 (0.2)	1.8 (1.22–2.64)	2.67 × 10^−3^	Not retained§		
Suspected diagnosis subgroup, Metabolic disorder	0.42 (0.2)	1.52 (1.03–2.24)	3.47 × 10^−2^	Not retained§		
Suspected diagnosis group, Inflammatory and autoinflammatory disease	− 1.01 (0.54)	0.36 (0.13–1.04)	4.55 × 10^−2^	Not retained§		
Department, Gastrointestinal and Liver diseases	− 1.2 (0.3)	0.3 (0.17–0.54)	1.90 × 10^−5^	Not retained§		
Setting, Medical day hospital	− 1.83 (1.08)	0.16 (0.02–1.34)	4.24 × 10^−2^	Not retained§		
Suspected diagnosis group, Pancreatitis	− 1.99 (1.07)	0.14 (0.02–1.12)	2.18 × 10^−2^	Not retained§		
Suspected diagnosis subgroup, Cholestatic disorder	− 2.47 (1.05)	0.08 (0.01–0.66)	1.41 × 10^−3^	Not retained§		

95% CI: 95% confidence interval; Beta: beta coefficient; SE: standard error

*Univariate logistic regression analysis

^†^Cox & Snell *R*^2^, 0.08; Nagelkerke *R*^2^, 0.11; Percent of cases correctly classified, 62%; AUROC, 0.637 (95% CI, 0.589 to 0.683)

^‡^Multivariable logistic regression analysis using the stepwise method

^§^Not retained in the multivariate logistic regression model

#### Predictors of a CES-guided treatment strategy in univariate and multivariable analyses

For 62 patients (14.6%), therapy was guided by their physician based on NGS results. In univariate analyses, several items were associated with a CES-guided treatment strategy (Table 7), and four maintained significance in multivariable analysis: ‘Confirmation of the suspected diagnosis, Yes or Contributive’ (OR, 3.75 [95% CI, 1.74–8.12]; *P* = 0.0008), ‘Suspected diagnosis subgroup, Hypercholesterolemia’ (OR, 3.45 [95% CI, 1.56–7.66]; *P* = 0.002), ‘Number of genetic variants reported in the CES report per patient’ (OR, 1.44 [95% CI, 1.06–1.97]; *P* = 0.02), and ‘Department, Reference Center for Inborn Errors of Metabolism’ (OR, 1.94 [95% CI, 1.02–3.69]; *P* = 0.04) (Table 7).

**Table 7:** Predictors of a CES-guided treatment strategy in univariate and multivariable analyses

Predictor	Univariate analysis			Multivariable analysis†		
	Beta (SE)	Odds ratio (95% CI)	*P* value*	Beta (SE)	Odds ratio (95% CI)	*P* value‡
Confirmation of the suspected diagnosis, Yes or Contributive	1.86 (0.36)	6.41 (3.16–13.01)	3.13 × 10^−9^	1.32 (0.39)	3.75 (1.74–8.12)	0.0008
Suspected diagnosis subgroup, Hypercholesterolemia	1.11 (0.36)	3.03 (1.51–6.08)	3.23 × 10^−3^	1.24 (0.41)	3.45 (1.56–7.66)	0.002
Number of genetic variants reported in the CES report per patient	0.5 (0.13)	1.77 (1.38–2.27)	8.24E × 10^−6^	0.37 (0.16)	1.44 (1.06–1.97)	0.02
Department, Reference Center for Inborn Errors of Metabolism	0.69 (0.29)	1.99 (1.12–3.52)	1.56E × 10^−2^	0.66 (0.33)	1.94 (1.02–3.69)	0.04
Setting, University Hospital of Nancy	1.74 (1.03)	5.68 (0.76–42.38)	2.57 × 10^−2^	Not retained§		
Number of genetic variants classified as LP or P per patient	0.59 (0.22)	1.8 (1.17–2.77)	8.62 × 10^−3^	Not retained§		
Number of genetic variants classified as VUS, LP, or P per patient	0.56 (0.15)	1.76 (1.31–2.36)	2.04 × 10^−4^	Not retained§		
Exome sequencing on the proband and at least one family member	0.64 (0.29)	1.89 (1.06–3.36)	3.39 × 10^−2^	Not retained§		
Suspected diagnosis subgroup, One-carbon metabolism disorder	0.64 (0.29)	1.89 (1.06–3.36)	3.39 × 10^−2^	Not retained§		
Suspected diagnosis group, Liver disorder	− 1.14 (0.61)	0.32 (0.1–1.06)	3.01 × 10^−2^	Not retained§		

*95% CI* 95% confidence interval; *Beta* beta coefficient; *SE* standard error

*Univariate logistic regression analysis

^†^Cox & Snell *R*^2^, 0.11; Nagelkerke *R*^2^, 0.20; Percent of cases correctly classified, 85%; AUROC, 0.768 (95% CI, 0.725 to 0.807)

^‡^Multivariable logistic regression analysis using the stepwise method

^§^Not retained in the multivariate logistic regression model

## Discussion

In this retrospective observational study of more than 603 consecutive subjects, including 426 probands, who underwent CES as a first-tier genetic test to investigate a Mendelian phenotype, we found a diagnostic yield ≈ 38% for reporting P/LP variants. The CES diagnostic yield was greater than 40% among patients with neurological disorders, dyslipidemia, developmental abnormalities, osteogenesis imperfecta, intestinal absorption disorders, or lipodystrophy.

Predictors independently associated with detecting at least one plausible P/LP variant included a CES prescribed in a pediatric care department, a suspected lysosomal disorder, or hypercholesterolemia. Regarding confirmation of the suspected clinical diagnosis, independent predictors were a CES prescription for hypercholesterolemia and the inclusion of at least one family member in addition to the proband. Finally, several predictors were associated with CES-guided therapy, including confirmation of the suspected diagnosis by CES, a CES prescription originating from the Reference Center for Inborn Errors of Metabolism, and the total number of genetic variants reported per patient.

Exome and WGS have been evaluated for use in genetic disorders, with more evidence for CES, exhibiting a molecular diagnostic yield ranging from 9 to 51% [2–29]. Overall, the utility of WGS in the genetic diagnosis armamentarium is unclear, mainly due to the lack of reliable data comparing it with CES in terms of diagnostic efficiency. One study of 108 patients suggested that WGS offers additional but limited clinical utility in this setting [30]. A recent study from UK 100,000 Genomes Project reported a diagnostic yield of 25% for WGS in a cohort of 2183 probands with a broad spectrum of rare diseases [20]. Interestingly, the diagnostic yield was higher among patients with suspected monogenic disorders (35%) than among those with complex phenotypes (11%) [20]. A systematic review published in 2021 reported the clinical utility of exome/genome sequencing across disease indications in pediatric and adult populations [31]. Among the 50 studies that met the selection criteria, the diagnostic yield ranged from 3 to 70%, with the highest yields observed in neurological indications (22 to 68%) and acute illness (37 to 70%) [31]. Interestingly, the proportion of VUSs ranged from 5 to 85% across studies, with higher rates observed in patients of non-European ancestry [31]. Our study identified three predictors independently associated with the CES diagnostic yield, including lysosomal disorders and hypercholesterolemia, which correspond to monogenic diseases and CES prescription from a pediatric care department. In this latter category, younger age at the time of disease presentation may be considered a surrogate marker of an extreme phenotype, thereby increasing the risk of a priori probability of a monogenic disorder. Furthermore, consistent with the results from UK 100,000 Genomes Project [20], the CES diagnostic yield in our study was > 40% among patients with osteogenesis imperfecta, developmental abnormalities, dyslipidemia, and neurological disorders, whereas it was < 25% among patients with more complex presentations, including immunodeficiencies and inflammatory diseases.

A limited number of studies have assessed predictors of CES or WGS utility among patients with various Mendelian phenotypes [9, 20, 32]. By evaluating 336 consecutive patients with hypertrophic cardiomyopathy, Bonaventura et al*.* reported the performance of the Mayo hypertrophic cardiomyopathy genotype predictor score, with a positive association between the clinical score and the diagnostic rate of an NGS-based panel of 229 genes [32]. An observational study in South Africa reported on the clinical utility of whole exome sequencing or targeted gene panel sequencing (207 genes) in 80 patients with suspected inborn errors of immune function [33]. Molecular diagnosis was obtained in 30% of patients (24 out of 80), of whom 67% had a significant change in management following molecular diagnosis [33]. A recently published Australian study investigated predictors of CES utility among 204 patients assessed in multidisciplinary renal genetics clinics and found younger age at presentation to be an independent predictor of CES efficiency [9]. Moreover, results from the UK. The 100,000 Genomes Project confirmed the highest diagnostic yields of WGS among family trios and families with large pedigrees [20]. In line with these results, including at least one family member in addition to the proband was independently associated with confirming the suspected clinical diagnosis by CES in our study.

Using a systematic approach and predefined outcomes, we explored the clinical utility of CES as a first-tier genetic test using a large dataset of consecutive adult and pediatric patients with various Mendelian phenotypes. Our results can be translated into several perspectives from a clinician’s point of view and a well-structured dialog framework between prescribing clinicians and molecular medicine physicians. First, the clinical utility of CES should be assessed in well-powered prospective studies using predefined inclusion criteria to allow for better inference of the results in a large population. In addition, a randomized trial should be designed to evaluate a first-line CES-based strategy in patients with high expected diagnostic yield compared to a first-line WGS-based strategy in patients with low expected diagnostic yield from CES, using a diagnostic yield cutoff ≈ 25–30%, as based on the results from UK 100,000 Genomes Project [20]. Second, in line with previous studies, our results reinforce the added value of family-based CES prescription, notably when VUSs are discovered [34]. In fact, the utility of an 11-gene NGS-based panel in patients with suspected hereditary hyperparathyroidism was evaluated in a 4-year retrospective study [34]. A pathogenic variant was identified in 16% of patients (19/121) [34]. Notably, a VUS was identified in 7% (8/121) of the patients, but two of the variants initially classified as VUS on the *CASR* gene were reclassified as LP after familial segregation studies and computational analysis. VUSs identified on *MEN1* and *CDKN1A* were downgraded to likely benign [34]. This study has highlighted the importance of re-evaluating VUSs to inform patient management and appropriate genetic counseling [34]. Current data suggest that 10–15% of reclassified VUSs are upgraded to LP/P, with the remainder downgraded to likely benign or benign [35]. In the context of CES and WES, an emerging consensus is to report VUSs only from genes with a well-established gene-disease association [35, 36]. The problem of VUSs will continue to grow with the expansion of genomic testing, although many initiatives are underway to improve the interpretation of genetic variants [35]. Third, our study shows that a CES prescription performed in a reference center is more likely to lead to a molecular diagnosis. Notably, confirmation of the suspected diagnosis by CES represented an independent predictor of CES-guided therapy. Our results support the importance of prescribing CESs in close interaction between referring physicians with expertise in genetic disorders and molecular medicine physicians.

We acknowledge several potential limitations of the study that should be considered in interpreting our results. First, we report findings from a retrospective single-center study, which need to be confirmed in independent cohorts [2]. Second, given the constantly evolving knowledge of gene and variant annotations, noncontributory CES may, in the future, reveal variants upgraded with respect to their pathogenicity [2]. Third, the efficiency of CES is suboptimal in detecting deep intronic or regulatory mutations and copy number variants (CNVs) [2]. In this setting, WGS exhibits an improved diagnostic yield compared with targeted gene sequencing panels, particularly by highlighting structural and nonexonic sequence variants not detectable by whole-exome sequencing [37]. Our study has several strengths. First, we report one of the most extensive European series of consecutive patients with a Mendelian phenotype and evaluate the diagnostic yield of a large CES panel in the routine practice of a Molecular Medicine Department. Second, we reported a median CES test-to-report time of 5 months, consistent with effective patient care. Third, in our bioinformatics analysis pipeline, we systematically combined classical methods of variant filtration and annotation with the SVS-PhoRank phenotype-driven computational algorithm, in line with our molecular diagnostic strategy based on gene captures that span several thousand disease-associated genes.

## Conclusions

In conclusion, using one of the most extensive series of consecutive patients with various Mendelian phenotypes, we evaluated the diagnostic yield of a large CES panel as a first-line diagnostic strategy in a real-life setting. Independent predictors of CES utility in terms of diagnostic performance and confirmation of the suspected diagnosis were criteria strongly suggestive of an extreme phenotype, including pediatric presentations and patient phenotypes associated with an increased risk of a priori probability of a monogenic disorder, the inclusion of at least one family member in addition to the proband, and a CES prescription performed by an expert in the field of rare genetic disorders. Our results can be translated into several perspectives from a clinician’s point of view and pave the way toward better CES prescription strategies to optimize CES diagnostic efficiency.

## Materials and methods

### French framework for the diagnosis and management of genetic disorders

As previously reported [2], the management of genetic disorders in France is organized around specialized reference centers within the “French National Plan for Rare Diseases” framework. One of these centers is located at the University Hospital of Nancy (ORPHA67872) and receives patients referred by their treating physician when a genetic disease is suspected. Genetic testing is performed after formal written consent from the adult patient or the proband’s parents in the case of pediatric patients. In the setting of the diagnosis of genetic disorders and inborn errors of metabolism, CES is 100% covered by the French health care system [2].

### Study design, setting, and patient selection criteria

We carried out a retrospective observational study on consecutive patients who underwent CES at the Department of Molecular Medicine at the University Hospital of Nancy (Nancy, France) as a first-tier genetic test to investigate a Mendelian phenotype. The inclusion criteria were as follows: i) CES prescribed between January 2016 and December 2019; ii) adult or pediatric patient; and iii) CES prescribed for a suspected genetic disorder other than phenylketonuria or hyperphenylalaninemia for which a dedicated molecular diagnostic approach is available via Sanger and/or targeted gene panel sequencing. There were no exclusion criteria. The study was observational, meaning that all clinical evaluations, biochemical investigations, imaging examinations, and clinical diagnoses were performed at the discretion of the treating physicians. The institutional review board of the University Hospital of Nancy approved the study.

### Data collected for the study

The following CES-related administrative and clinical data were retrieved through electronic chart review using DxCare® software (Dedalus France, Le Plessis Robinson, France) for patients followed at the University Hospital of Nancy or medical prescriptions for patients outside the University Hospital of Nancy: institution (university hospital, regional hospital, or private practice); setting (outpatient clinic, hospital department at the University Hospital of Nancy, medical day hospital, or outside the University Hospital of Nancy); adult or pediatric department; geographical region; department of CES prescription; proband vs. sibling; trio analysis (yes/no); sex; date of birth; date of CES prescription (age at CES prescription expressed in years); date of CES clinical report; date of the molecular diagnosis report established by the molecular medicine physician (test-to-report time expressed in months); and suspected diagnoses, which were regrouped and classified into main categories based on recruitment by the Department of Molecular Medicine and the Reference Center for Inborn Errors of Metabolism (metabolic disorder; dyslipidemia; liver and biliary tract disorder; neurological disorder; inflammatory and autoinflammatory disease; developmental abnormality; mitochondrial cytopathy; pancreatitis; intestinal absorption disorder; myopathy; osteogenesis imperfecta; lipodystrophy; primary immunodeficiency; and miscellaneous conditions). CES-related genomic data were extracted from the molecular diagnosis report established by the molecular medicine physician and included the following: number of retained variants; variant annotation of each genomic variant (#1 to #4 in the order reported in the CES report) according to Human Genome Variation Society nomenclature using both coding DNA (c.) and protein (p.) reference sequences; gene; variant classification according to the American College of Medical Genetics and Genomics (ACMG) criteria [38]; and patient genotype status. We assessed follow-up data for each patient with regard to the clinical utility of CES as reported by the prescribing physician: 1) confirmation or contribution to the clinically suspected diagnosis and 2) treatment adaptation based on the conclusions of the CES report.

### Next-generation sequencing

We performed CES using TruSight One Panel and the Illumina MiSeq platform (Illumina, Evry, France) or TruSight One expanded panel and the Illumina NextSeq 550 platform, in compliance with the French Accreditation Committee requirements, at the University Hospital of Nancy and the Functional Genomics Facility of the INSERM unit UMR_S 1256 (NGERE; UMS2008/US40 IBSLor), as previously described [2, 39, 40]. At the Department of Molecular Medicine at the University Hospital of Nancy and the National Reference Center for Inborn Errors of Metabolism, we opted for a strategy based on Illumina TruSight captures that cover a large number of disease-associated genes instead of a full-exome-based approach. TruSight One Sequencing Panel provides comprehensive coverage of > 4800 disease-associated genes; TruSight One Expanded Sequencing Panel targets ~ 1900 additional genes with recent disease associations in the scientific literature (Additional file 1: Table 1). We used Nextera and Nextera Flex enrichment solutions with TruSight One and TruSight One Expanded captures, respectively. TruSight One Expanded and TruSight One cover 100% and 82% (145/176) of the 176 mitochondrial nuclear genes reported in Additional file 1: Table 1. Our bioinformatics analyses are detailed in the Supplemental Methods.

### Study aims and outcomes

The aims of the study were as follows: i) to assess CES efficiency for achieving a molecular diagnosis as a first-tier genetic test in patients with a Mendelian phenotype in the entire cohort and according to the suspected diagnoses; ii) to assess predictors of discovering at least one variant classified as pathogenic or likely pathogenic (LP) (P/LP); iii) to evaluate predictors for confirming the suspected clinical diagnosis; and iv) to explore predictors of a CES-guided treatment strategy by the prescribing physician.

The study outcomes were the discovery of at least one P/LP variant, “Confirmation of the suspected clinical diagnosis by CES”, and “CES-guided treatment strategy”. In diagnostic yield analysis, we considered patients for whom the CES report retained at least one genetic variant classified as P/LP or by using a broader definition by adding patients with at least one genetic variant classified as of uncertain significance (VUS). Assessment of “Confirmation of the suspected clinical diagnosis by CES” and “CES-guided treatment strategy”, as outcomes, was based on chart review and the medical consultation report prepared by the prescribing physician following the molecular diagnosis restitution to the patient or the parents’ proband.

### Statistical analysis

Categorical variables are summarized as frequency counts and percentages with a 95% confidence interval (95% CI). Quantitative variables are expressed as medians and interquartile ranges (IQRs, 25th and 75th percentiles). We used univariate logistic regression to identify predictors of i) discovering at least one variant classified as P/LP, ii) confirming the suspected clinical diagnosis, and iii) a CES-guided treatment strategy by the prescribing physician. To explore the variables independently associated with the outcomes studied, all significant variables from the univariate analyses were included in a multivariable logistic regression model. All variables with *P* < 0.05 were retained in the model. Results are shown as odds ratios (ORs) and 95% CI for each independent predictor and the percentage of cases correctly classified by the logistic regression model. We assessed model discrimination using ROC analysis and the percentage of cases correctly classified by the model. We assessed the goodness of fit of the model using Nagelkerke R^2^ and Cox & Snell R^2^ statistics [41]. All statistical analyses were conducted using MedCalc, version 19.5.3 (MedCalc Software, Ostend, Belgium) and SVS (v8.8.1; Golden Helix, Inc., Bozeman, MT, USA).

## Supplementary Information

**Additional file 1.** Supplementary Appendix (Supplementary Methods, Supplementary Tables, and Supplementary Figures).

## Data Availability

Anonymized patient data are available for use in collaborative studies to researchers upon reasonable request (abderrahim.oussalah@univ-lorraine.fr). Data will be provided following the review and approval of a research proposal (including a statistical analysis plan) and the completion of a data-sharing agreement. Responses to the request for the raw data will be judged by the IRB of the INSERM UMR_S 1256 and the University Hospital of Nancy.
