# Supplementary material for: Predictors of the utility of clinical exome sequencing as a first-tier genetic test in patients with Mendelian phenotypes: results from a referral center study on 603 consecutive cases
Source: Hum Genomics. 2023 Feb 5;17:5. doi: 10.1186/s40246-023-00455-x (PMC9899384; doi:10.1186/s40246-023-00455-x)
Supplement: Supplementary file 1 — Additional file 1. Supplementary Appendix (Supplementary Methods, Supplementary Tables, and Supplementary Figures). [file 40246_2023_455_MOESM1_ESM.docx]

**Predictors of the Utility of Clinical Exome Sequencing as a First-Tier Genetic Test in Patients with Mendelian Phenotypes: Results from a Referral Center Study on 603 Consecutive Cases**

Tom Alix, PharmDs, MScs^1^; Céline Chéry, PhD^1-3^; Thomas Josse, MSc^1^;

Jean-Pierre Bronowicki, MD, PhD^2,4^; François Feillet, MD, PhD^2,3,5^;

Rosa-Maria Guéant-Rodriguez, MD, PhD^1-3^; Fares Namour, MD, PhD^1-3^;

Jean-Louis Guéant, MD, DSc, AGAF^1-3^; and Abderrahim Oussalah, MD, PhD^1-3^

**SUPPLEMENTAL METHODS**

**2.4. Next-generation sequencing: bioinformatics analysis**

Bioinformatics analysis was performed as previously described (1-4). The Burrows-Wheeler Aligner (BWA) software package was used to map all reads to GRCh37/hg19 annotation using the BWA-MEM algorithm. PCR duplicates were removed from alignments using the Picard (MarkDuplicates) tool (http://broadinstitute.github.io/picard). Indels were realigned using the Genome Analysis Toolkit (GATK) (5). SNVs and short indels were called using the GATK HaplotypeCaller software (5). We used the following strategy for filtering and prioritizing the genetic variants: Step #1: Import all retrieved genetic variants; Step #2: Retain only low frequency and rare variants by excluding variants with a reported alternative allele frequency (AAF) >5% in the following databases: The Exome Aggregation Consortium (ExAC) and the Genome Aggregation Database (gnomAD Exomes). The aim of step #2 is to retain rare (AAF <1%) and low-frequency (AAF <5%) variants that exhibit relatively large effects on disease risk, such as those involved in severe monogenic diseases (6); Step #3: Exclude intronic variants other than splice donor, splice acceptor or splice region variants or synonymous variants; Step #4: Prioritization of genetic variants for their association with the studied phenotype using the SVS-PhoRank gene ranking algorithm based on the Phevor algorithm (6). The Phevor algorithm scores ontology terms based on their proximity to patient’s phenotypes. The SVS-PhoRank score was calculated according to the RefSeq Genes 105v2, NCBI gene source, using the following ontologies: Human Phenotype Ontology, Gene Ontology, and OMIM Phenotype Ontology. Functional variant annotation was performed using the following tools: MutationTaster (7), Functional Analysis through Hidden Markov Models (FATHMM) (8), MetaSVM / MetaLR scores (9), Sorting Tolerant From Intolerant (SIFT) (10), Protein Variation Effect Analyzer (Provean) (11), and deleterious annotation of genetic variants using neural networks (DANN) (12). Variant pathogenicity was reported according to the ACMG criteria (13). All retained and top-prioritized variants were systematically assessed for their ClinVar annotation (14). All the genetic variants retained in the molecular diagnosis report had to meet high-quality metric criteria (read depth [RD] >20, alt-read ratio [ARR] >0.2, genotype quality score [GQ] of 99). All the genetic variants retained in the bioinformatics analysis were confirmed by visual inspection of the read-level data of BAM files. Sanger confirmatory sequencing has been performed in rare cases of variants with suboptimal quality metrics indicators (e.g., splice donor or acceptor variants, homozygous variants). Large structural variations were not assessed. All bioinformatics analyses, including the SVS-PhoRank gene ranking algorithm, were performed using the SNP & Variation Suite (v8.9.0; Golden Helix, Inc., Bozeman, MT, USA).

**SUPPLEMENTAL TABLES**

**Supplemental Table 1. List of genes included in the TruSight expanded and the TruSight one panels and their overlapping with mitochondrial nuclear genes**

| **Panels** | **Total** | **Detailed list of genes** |
| --- | --- | --- |
| Mitochondrial genes*  AND  TruSight expanded†  AND  TruSight one‡ | 143 | *NDUFS6, ABCB7, SLC25A3, COX15, DGUOK, COQ9, NDUFS4, ELAC2, POLG, PDP1, COX10, L2HGDH, ACO2, OPA3, ETFA, ISCU, FOXRED1, NDUFAF1, UQCRQ, TUFM, AUH, NDUFV2, FARS2, TK2, NDUFB3, FH, SLC25A4, D2HGDH, TMEM126A, TAZ, MTO1, PDHX, PANK2, NDUFA12, BOLA3, HARS2, MARS2, TSFM, NDUFS3, NFU1, TYMP, DARS2, AIFM1, POLG2, ACACA, YARS2, NDUFAF6, COA5, PUS1, OXCT1, EARS2, NDUFS2, UQCRB, AMPD1, AGK, LRPPRC, COX4I2, SUCLG1, COQ6, MRPL3, COX6B1, NUBPL, HIBCH, SURF1, PC, SLC25A19, TIMM44, RRM2B, NDUFA9, SLC25A12, DLAT, LARS2, ATP5E, PDHB, ATPAF2, GARS, PNKD, NDUFB9, PDHA1, NDUFS1, NDUFAF4, ALDH3A2, PCK2, NDUFA2, FXN, OPA1, C12orf65, PDSS1, RARS2, BCS1L, APTX, FASTKD2, SDHAF1, GCDH, TIMM8A, TTC19, LIAS, TMEM70, AASS, NDUFV1, XPNPEP3, NDUFA1, MRPS16, MPV17, GFER, SCO2, ETFB, COQ4, DNM1L, SCO1, NDUFAF3, COX14, SARS2, NDUFA11, AFG3L2, NDUFS8, IDH2, SLC19A3, MTFMT, CHAT, GFM1, MRPS22, GAMT, TWNK, MTPAP, AARS2, SACS, ACAD9, ETFDH, NDUFAF2, ETHE1, TPK1, ABAT, NDUFAF5, DLD, TACO1, PDSS2, NDUFA10, SUCLA2, NDUFS7, COQ2, TRMU, DNAJC19* |
| TruSight expanded†  AND  TruSight one‡ | 4590 | *CLMP, C5orf42, SLC18A1, RPS17, MMP2, CD44, ABCA3, TMEM216, PAX7, KCNMA1, A4GNT, ZHX3, ERCC5, MEFV, XDH, GCSH, EDN1, CYP11A1, ABCC6, STOX1, DECR1, CXCR4, SALL1, NCF4, NAGPA, HYMAI, IRAK1, CFHR5, LMAN1, LDB3, IFT122, GALNT3, CHD8, SUMO1, GP1BA, NTRK1, LRRC6, ANGPTL5, SLC6A11, TOP1MT, MYO9B, CNGB1, MMP7, EP300, GPSM2, TBX10, PRKCG, NOS2, TRIB3, DTNBP1, DDX5, SLC26A1, COL4A5, ZEB1, SOD2, CCDC28B, MSRB3, KCNK6, XK, MET, BANK1, ADAM7, MAX, TNS3, NR4A2, MED13L, EVC, TEAD1, SORL1, NFIA, SYN3, LEP, UBA1, SLC22A2, DMC1, SERPINF1, TP53AIP1, ATXN10, NMNAT1, HMX1, DIP2A, CCL22, LHB, PTPN22, CYP2F1, ITGA3, RABGGTA, BRCA1, SLC26A10, CYP2D6, CRYGC, RUNX3, EFNB1, PRKCB, LIPN, ACSM3, A2M, SLCO2B1, LMNB2, ABCD4, ROCK2, BBS12, VKORC1, ELANE, TEK, TRPM6, UGT2B7, FEV, COL1A1, SLC8A1, PDCD5, GRK5, CLDN16, AP1S2, ISL1, NUDT1, GRK1, PRDM2, CD1A, FOXP3, SMYD3, SELL, WDR45, NEIL1, ANO5, LTBP4, GFPT2, RBM10, PYY, ARHGEF9, GPR55, MFF, RAD23B, HIST3H3, PCDHA13, FOXE1, CNGA3, CD38, NAT8L, NEFH, MFSD8, ALX1, ROR2, GSTA2, SMUG1, ZNF385B, MAOA, APOE, TNNT3, GYPC, NR2E3, RFXAP, C6orf15, ALG2, PLEKHG4, TPM3, EPB42, HSPD1, PIWIL3, F2RL1, PRY, NTHL1, OPHN1, TRAPPC9, ADH5, SHBG, KIAA0100, MYH6, LIM2, BCHE, GCLM, SLC24A5, BCL2, HCRTR2, HBZ, ARL13B, CAMTA1, ORC1, DRD3, AIPL1, SNX19, NME8, CHFR, THADA, PIK3CB, COL18A1, PXDN, TNXB, A4GALT, CARD8, FUS, EMX2, TOX3, TF, RSRC1, COG1, THRB, MYBPC1, NPR1, MTMR14, DNAH11, ASS1, AQP2, ALAD, SLC9A9, GSTM4, F7, RSPH4A, TFR2, RAG1, IRS1, SRP72, TSPAN7, POMT1, MFN2, LRRC8A, CYP1A1, ATR, THAP1, GBGT1, GRIK2, PCDH18, GNRHR, CHST6, DPYD, HTR3A, POF1B, MAPK10, COX7A2, QKI, KDM6A, NCAM1, MAN2B1, OFD1, ACADL, FERMT3, ABHD12, AGT, SLC22A14, GLIS2, CGA, PEX16, FGF1, IL6R, CHRM1, CHRNB2, UBN2, COL7A1, SEZ6, DBH, TFAP2B, CIITA, MUTYH, FANCC, RP9, NQO2, CDH8, AGPS, NEU1, PCCB, UNG, SPRY2, PRSS56, LZTFL1, XPC, KLHL7, GSTP1, CCR3, SLC9A3R1, HBG2, ASAH1, ADRA1A, ZNF350, PTPN2, GIPR, MIR206, BST1, AHI1, SLC3A1, KISS1R, GORAB, GFI1, FHL1, GATA1, LPIN3, AHR, MYLK2, MAD2L1, PRPF3, ATP10A, CHMP2B, TAS2R3, NFE2L2, IL23R, SPINK5, CYP4A22, UNC5CL, KLKB1, HP, BTK, SYTL5, PRND, PIGL, IDS, HS1BP3, CLCA1, UIMC1, FXYD2, ZCCHC12, ATP7B, TRB, DCX, TBX21, PPARGC1A, KCNJ6, CASP5, OTOA, AHRR, POLRMT, NDUFA13, CYP19A1, CD151, ZAN, CRYBA1, NPFFR2, HRH2, SOX9, CYP3A5, CUL4B, CENPJ, PITX1, RDX, CARD9, SLC34A3, CATSPER1, OAS1, CHRNB1, ADCY3, DCTN1, LIN28B, CDY2A, ERMAP, GRIP1, APBA2, MUC2, TCF21, JPH2, AMELX, C5, SLC2A10, ANKRD26, ATP2B2, TCP1, SEPT12, CMA1, HCRTR1, PAWR, TLK1, LDLR, ASTN2, ELAVL2, TAB2, SIX2, GPATCH8, HPS1, FAM161A, PGM1, SNCAIP, KCTD13, HLA-DOA, CSMD3, RCAN1, IRF7, UGT1A8, PECR, FAM120A, SHH, SH2B1, THPO, TYRP1, CD177, JAK2, TMPRSS4, TSHZ1, BICD1, TNFRSF1A, AKAP2, USP9Y, CASP1, CD8A, ACADVL, GTF2H5, MYCL, MIR17HG, TNNT2, PLA2G4C, ABCG8, PHGDH, JRK, XIAP, GPS1, APOA2, KALRN, VAX1, IFNAR1, ATP6V0A2, SLC29A3, TALDO1, SDHD, USH2A, SUMF1, B2M, NF1, MIRLET7E, IMPAD1, OLR1, NEGR1, EHD2, NCF1, SPG7, AGER, CBS, MICA, GPR33, SNTA1, IL20RA, ELF4, CACNG2, TLR5, H2BFWT, KCNV2, CLPS, BTBD9, NT5E, NCSTN, GALNT9, NPY, IL18, GCGR, SLC16A2, NOTCH2, ADD1, KDM6B, CLEC4M, MGP, SOST, GYPA, ACLY, CYP4F12, KIAA0232, FKBP5, SMS, ERCC8, PARD6A, GPX4, GCH1, TYMS, SCNN1B, ZNF750, MCM5, ALG9, CSNK2A2, CPA4, MEGF11, CUL3, KRT74, SNCA, ITK, ESRRB, PRCD, HRAS, RABL6, MAP2K4, ITGB2, CD72, TEKT2, CDKN2B, ZNF480, PROS1, PSMB8, MAP6, XRCC1, VEGFA, LAMA1, APOL1, SETBP1, ZBTB41, ITGA2, MME, SHANK3, GRIN2A, MAST4, EEF2K, CCDC14, COG8, MYC, NRXN1, ASIC3, ARSA, SUPT16H, PHYH, SOCS3, UNKL, CCNA2, IL10RB, ARID1B, WNT10B, IL1RAPL1, WDPCP, VCP, PCSK9, NKX2-1, SLC35A1, LAMA3, SLC14A2, COCH, SLC30A2, SCGB1D2, SLC11A1, SH3GL1, SLC25A39, EIF3H, KIF1B, GSK3B, GPD1L, KIR2DL1, TNFAIP3, PLA2G10, ADAMTS17, BLK, TAAR9, CLCNKB, APOM, PKP2, SPATA16, AMPD3, NPHP3, PTCSC3, TSEN54, MMP1, FBXO7, CA12, PDE6G, HLA-DQA1, STH, GCLC, ZC3H14, CBX2, MAN1A2, ALDH5A1, FKTN, CTDP1, CRTAP, ZNF75D, IL18RAP, KRT86, STMN1, DPP6, MKKS, STK39, CD109, SFTPA1, RHO, GRID1, TP53I3, BANF1, CR1, SLC22A3, HMBS, EBP, TOR1A, P2RY4, TRPV1, SLC25A15, GADD45B, UCP2, TDP1, FOXG1, WFS1, RAD21L1, PROC, PABPN1, SLCO1C1, TAAR6, SLC22A5, GRIN3A, FPR1, ATL1, ASPN, SMOC2, SERPINB11, NDE1, PPARD, FSHB, HLCS, AK1, CLNK, TBC1D24, STK10, LGALS2, AAAS, ABCD3, C4B, DGCR8, GSR, NEU2, CACNA1C, FIG4, IGHMBP2, SP7, BHLHA9, CCL11, IDO1, OBSL1, SLC23A1, SLC39A4, CITED2, GRM3, FCGR2A, RBMY1A1, GPIHBP1, KRT9, FOXP2, HTR1A, NLGN4X, ALOX12, CCDC103, RB1CC1, POLR3A, NEIL2, EIF4E, ABHD5, EXOC4, GSC, TRMT1, KMT2D, IMPG2, SLC29A4, MMP3, CRHR1, TRAF6, AXL, LMNB1, PNPLA3, F13A1, SLC10A1, CSNK1E, BRCA2, ACADM, KCNQ4, FMR1, FAM8A1, TUBB2B, CXCR1, BBS9, USP15, SLC12A3, C9orf72, PTGER2, SLC5A7, ERCC4, SLC31A1, MMP8, ESAM, ACY1, RNASEH2C, PKN3, COMMD1, CBX4, BLMH, NTRK2, ADCYAP1, MFGE8, SP110, KIAA0513, SH3BP2, SH3TC2, IL10, DHH, AGRN, CEP290, CNPY3, RTN2, TFB1M, PRPH2, IRX4, DXO, SPANXN5, NFKBIL1, TLR10, CHD7, APBB3, WDFY3, SLC30A8, CEP63, RPS10, DPM1, IAPP, KLK7, ALOX5AP, LRP1, NKX2-3, GP1BB, ANKH, RIPK3, TAS2R9, RFT1, RAET1L, SLC6A1, SH3PXD2B, SAMHD1, OCLN, EPC2, ATP13A2, NELFA, NAGLU, PAX6, KRT4, SCG2, CDON, TREM2, SERPINI1, MMP14, FMO3, POU4F3, MDM2, KCNK9, TAP2, CALHM1, THSD7A, NBEA, LHX4, AKT2, EGFR, MAP3K15, NDRG1, PTPRQ, GBE1, FMO6P, SNORD50A, SOD1, SIRT5, BCR, ZFP36L1, PAPD7, CYSLTR1, NKX3-1, CHD2, TRIM17, GALP, ERBB2, CREB1, FANCM, TRAK2, DDX3Y, LGALS3, UGT1A7, MYT1L, CFL2, TMPRSS3, UPB1, FUT3, KCNA3, CLDN14, DBI, PIF1, LPIN2, CDT1, SMARCA4, DIABLO, TRHR, TPSB2, LRP8, TGFBRAP1, MASP2, CNTN1, AGL, BBS4, MINPP1, CCL3L1, TMEM43, HS6ST1, AKR1D1, STAT5B, PRPF8, SCT, SKI, OAS2, GLIS3, MPP6, TBL1XR1, KRT16, G6PD, KLF5, TNFSF8, NUDC, PROKR2, VCX3A, TACSTD2, HBE1, BCAT2, C5AR2, NRG3, FOXI1, MYOC, F10, BCKDHA, GIF, COMP, THBS1, TAL1, TCAP, PLEKHG5, SNAPC5, MOCOS, VIP, SLC6A8, CALCR, MC2R, TBX3, CHRFAM7A, VCY, DIO2, MPLKIP, PRX, RAD50, TRIOBP, CABIN1, SYNE1, MTHFD1L, MBTPS2, MVK, TUBGCP4, TSPYL1, UVSSA, NCOA1, CDK7, CRELD1, CHEK2, GAS2L2, DRD4, ADH4, CD247, CDAN1, GRPR, SYT11, MCM4, DCAF13, SPTBN2, HTRA1, SLC26A2, ATP2B4, SPATA31C1, PTH, TARDBP, SLC24A1, LRSAM1, B3GALT6, CETP, APOC2, NXNL1, PTPN6, HADHB, ADAR, P2RX1, ILDR1, ARMS2, HBM, GRIK3, PDE10A, GRXCR1, CACNB2, EN2, TCTE3, RHOG, HBB, HEY1, KNG1, CASR, GPAM, CANT1, IRF2, RUNX2, PGR, GRK4, SRPX, PRPH, FGF10, ODC1, SDC3, MC3R, PHEX, SOX6, MSMO1, THRA, POLH, PTGES2, ENO3, COL4A6, GAS1, SGSH, SLC35G2, CXCL10, TRPM3, GALT, PLG, FECH, TYR, ATP6AP2, TACR3, SLC7A7, CA8, SLC35C1, IL10RA, KIF1A, IL36RN, VCAN, UBE2A, TSLP, NHS, CNTNAP5, SLC1A3, RNF139, SFTPC, RPL35A, SHANK2, MIR140, JUP, SLC41A1, LRCH1, RBMXL2, SULF1, PRDM5, DCXR, RAB11FIP5, TTTY13, SOHLH1, CDKN1A, LPL, MAGEE2, CLEC2D, HCN4, HFE2, CUL7, LIPC, DIO1, EIF2B1, GSTZ1, SLC6A12, RASA1, ITCH, ZCCHC13, GATA2, SLC2A2, EXO1, HRC, GABRA5, PIK3R1, GPC3, TAF1C, MAF, PDGFRA, PTPRJ, CSTB, TET1, ADRB3, DMD, FAM205A, MS4A12, FPR2, OTC, FOXH1, AGMO, KCNQ3, ABCB11, SLC22A12, FZD3, TMIE, HADHA, CCBE1, DLG5, SAGE1, PER1, ZFYVE27, SMARCAD1, BMI1, VMA21, MASP1, SLURP1, SHOX, ATRNL1, MIA3, AREL1, TMEM135, G6PC, CHRM3, TBX22, TOMM40, CPE, UPF3B, TUBGCP5, NOBOX, ADNP, POLD1, VIPAS39, BTD, LCAT, MIR196A2, APOA4, POMC, PMS1, IL9, HOXA11, RAB28, TFCP2, NLRP12, ALAS2, ST3GAL5, RPN2, NFKBIA, CNOT3, MIR2861, IGFBP3, GAK, PSMA6, ARHGAP24, ALB, C4BPA, CPT1A, C1QA, ALMS1, KIF22, DCN, GSE1, PRB4, NINJ1, EFR3A, COL11A1, UCP3, FOXF2, MLH1, VAMP7, IFNAR2, HLA-C, CYB5A, SCN10A, NXF5, MKS1, P2RY11, CX3CR1, ITGAE, PLP1, KISS1, ZNF804A, FUT2, PTCHD1, FZD4, NARS2, GALNS, TMLHE, SERPINB3, LIN28A, KCNJ11, FAM20C, TSPAN17, ABCC8, BPI, DHRS4L1, BNC2, SERPINA3, XKR4, MCPH1, MT2A, FABP2, SYNM, BMP2K, ALG1, TBC1D23, HEXA, HNRNPU, MAP2K3, FLT4, SCAP, OMG, NDP, SLC4A4, FKBP6, BRAF, TWIST1, EPO, PAX5, GJB4, COL1A2, DTNA, LY96, AANAT, AKT3, CRB1, KLK3, IL1R1, LTBP3, KHDC3L, AMHR2, DDX20, PROCR, PHKB, PHF11, PASK, HEPACAM, KMT2C, SI, GGT5, KCNE4, TRIM5, KCNS3, APOC1, BAAT, ARID1A, MBD1, MLYCD, FLVCR2, ZBTB40, AP1S1, SDHA, CYP4B1, COL12A1, ALPL, SMAD6, CHST3, SAT1, BTAF1, COLQ, NRXN2, UGT2B28, HYAL1, ABCA13, PNP, HDLBP, RFX2, MAPK8IP1, CHRNA7, PIGM, GNB3, FANCD2, ARL6, MEF2C, PSPH, LBR, IL2RG, AFF3, COG7, MYB, XRCC2, RPTOR, ERBB4, KANK1, ZAP70, GPHN, DNASE1, SLC6A2, LPP, PTPRCAP, IL12RB1, ATRX, DMRT1, CHST8, ACTN3, ELP4, RYK, IRS4, HSPB6, NMB, GSTA1, MIR146A, TGFB2, SEMG1, TRAF3IP2, CHRDL1, CHRNE, LAMA2, HSPB8, RLBP1, GAL3ST3, AXIN1, ARVCF, TRIO, POLR1C, NID1, ID3, KIF7, C1QC, MMP13, C8A, MTM1, CAPN3, CSNK1A1L, HCP5, RNF170, KBTBD13, KCNE1, CD4, DSP, NDST1, IRF1, FAM20A, SDHB, CLRN1, MBL2, SH2D1A, MUSK, TPP2, PLIN4, GTF2E1, PKD1, JAM3, DDOST, CHSY1, DIRC2, MYOM1, PHKG2, EXT2, DIAPH2, DOLK, ACVRL1, CLN8, MECP2, FCGR2B, DIP2C, NFATC4, NOS1, SNIP1, BMPR1A, YBX2, PRL, TIMP3, RFWD2, CIB2, ABCG1, LRTOMT, ARL11, SPAG16, CREB3L3, COG6, TMEM127, ARHGEF12, RAC2, GLUD2, CD55, PLN, PRDM9, SMAD3, TBX5, RHD, HMOX2, PTGDR, GJB6, CCDC39, ZNF513, PLP2, HTR2C, MDH1, ITGB4, KRT13, CACNA1E, PEX14, FMO4, PAFAH1B1, OTOR, DRD5, UBE2I, FYCO1, FBLIM1, PLTP, MUC3A, DUOXA2, CNTN4, ANGPTL3, MYO6, DNAJA4, DOCK8, EZH2, MYO1C, FAS, KIFAP3, PON1, SPRN, HBEGF, OSMR, SOD3, CYP21A2, CYSLTR2, ZC3H3, DDAH1, ACBD5, MGAT1, ALDH4A1, UGT2B15, MIR502, DNASE1L3, CRYAB, PRY2, SLC26A3, KIF11, SLC40A1, PTHLH, TNFRSF11B, PKLR, OR7D4, MIR27A, CD36, EXO5, OPN4, ARF4, OR13G1, CLK2, ZPBP2, LMF1, TNFSF15, FANCL, NCF2, SECISBP2, SLC12A6, MRPL48, XRCC6, GABRR2, HUS1B, PIK3C3, DHDDS, PAX9, NEFM, C1QTNF5, BCL9, SYN1, ABO, PIGV, TRAF3, LETM1, APBB1, NIPSNAP1, F9, LIPE, B4GALT1, CNGB3, CHD3, BCAP31, CHD6, HMCN1, CD40, FGD4, GDI1, ZNF81, PDXK, CCM2, EFTUD2, TAP1, LRRC4, CYP11B2, IGFBP7, FCRL3, SCN4A, FGB, EHMT2, EPHA5, OVGP1, FOLR1, HACE1, MYOT, HAL, BCL2L2, EMG1, AQP4, MERTK, RD3, SLC47A1, ORMDL3, TBXA2R, ALOX15, DMPK, TECR, SMC1A, CCL7, TP73, DSC2, GRIK4, TMPRSS6, ATP13A4, SERPINA1, ANTXR2, COL4A3, SLC6A18, PEX1, TAPBP, RET, MASTL, RUNX1, FADS2, IL1RL1, ALX3, KIR3DL2, MID1, SNX10, LIPH, ATP2C1, CYP4F3, ALDH1A2, GABRG1, AHCY, ALG12, CCL17, KHK, ESRRG, CNGA1, RDH5, ISPD, GLP1R, CD58, CSTF2T, TRIM37, TMEM8A, IFNL3, GRHL2, SELE, NLGN2, PROKR1, ABCB1, CNR1, GPR12, ABCC4, DLG3, MFRP, CC2D1A, STRADA, PLAGL1, MAGT1, NR2E1, MS4A6A, LLGL1, FTL, KLF6, THBD, NR5A1, SRR, CYP4V2, CHKB, HUWE1, FAAH, IL17A, TMPO, ACCS, LEPR, LTBP2, SV2B, CYP2A6, PGBD1, GP6, SLC25A22, ILK, SLC22A1, EYA1, PITX2, MYBPC3, GJA4, FABP3, RNF113A, MYPN, CAMKK1, SIM1, NHP2, SATB2, CLCN2, KCNJ9, ZFHX4, DPM3, AP4B1, SLC25A13, SMPD1, TH, COL6A2, ANKS1B, MFSD2A, CHL1, VANGL2, CNTF, AKAP10, FA2H, ALG13, KLHDC8B, TRD, SLC13A2, GSN, EGR2, WWC1, FRMD6, MYH3, SULT1A1, HOGA1, ANK2, RAB23, PLA2G4A, AKR7A2, FKBP10, VLDLR, CERKL, MAP2K2, DBT, SLC1A2, INVS, MPO, ZBTB16, PDGFB, ELN, NTF4, VIM, MPI, TLR8, HPS3, ABCC3, HLA-DMB, SPR, HOXA10, KIF21A, RHAG, LZTS1, CLUL1, CPN1, HLA-A, RSPO4, LDHA, EDNRB, SLIT3, CD27, PEX13, EGLN1, MIR106B, GRIN2B, NR1H2, NLRP7, CD226, TCTN1, STAT3, WDR36, TPCN2, MBD4, ROBO1, ATN1, ARSB, ORAI1, TSSK3, RUVBL1, ADAM9, TPH1, MAD1L1, APAF1, UBA3, NEK1, MYH11, MATN3, IFNGR2, NDUFA4, ANKRD11, ITPR1, SEZ6L2, SEPT9, DDAH2, GUCY2D, ATXN3, CIDEA, SLC26A4, SLC45A2, H19, SOX17, PAK3, DPYS, TGFBR1, PWRN1, WDR4, HK1, VPS33B, NAA10, HEXB, ACVR1C, NDUFAF7, MIR24-1, GALNT12, POMGNT1, TNKS, CRLF1, ABI3BP, PYCR1, GRHPR, WAS, MADD, ATM, LTN1, HSPA1A, BLOC1S3, TINAG, NEUROD1, IL17RB, GNAS-AS1, PARP2, GSTT2, CAMP, ADAMTSL2, PPIB, CYP2A13, UGT1A4, GOLGA5, SMPX, ALDH7A1, LCA5, KCNJ10, DHX37, UGT1A6, ANG, ACSL5, SMAD4, NMT2, SLC6A20, RRP1B, PFKM, EEF1B2, STAT6, DKC1, CCDC40, PER3, DAZ1, SCARB1, CACNA1F, UBD, DNAJB6, NAGS, GREM1, SUN2, PRKAG2, AGXT2, RFX8, KERA, PPT1, MLLT3, UBE2B, NUMBL, MUC4, MST1, PLOD3, SULT4A1, HAVCR1, BPY2, C7, WASF3, DYNC2H1, GLYCTK, MTUS1, GABRA6, UROS, MMEL1, TRAPPC10, LEMD3, MGMT, AIF1, MR1, FFAR1, CSH1, MAP4K5, F8, ACHE, SLC28A1, KRT6A, EGR3, MYO7B, SERPIND1, PTF1A, IFRD1, LCE3B, SIGMAR1, MTNR1A, PPP1R3A, MCL1, PABPC4L, PRNP, ACTN2, SHMT1, ADCY10, FOXA3, NEB, KLK15, TLR3, IL6, CTGF, FGF14, POLB, BRD1, PRICKLE2, ZNF335, MTMR9, MCCC2, CPT2, IVD, NSDHL, UGT2A1, EPOR, TOPORS, CLDN19, CASP3, CACNA2D3, CCRL2, RNF213, TMEM67, SLC7A2, NCKAP1, HSD3B7, VDR, ACE, AGO1, TAS2R16, IRF4, OPN1SW, ZNF213, ITGAM, L1CAM, DSPP, RGS2, MST1R, DLL3, FASLG, COL3A1, MIR126, CACNA1D, ETNPPL, PRKAA2, ARHGEF11, UHRF1BP1, PIK3R4, NDUFV3, UBE2NL, KRT14, PACRG, BBS2, ABCG2, PGAM5, GRB10, TBL1X, PCDHA10, SPAG17, HNF1A, EPHA3, NIPSNAP3A, INSIG2, STXBP2, FUT1, PECAM1, MS4A1, TMEM9, PTPN1, PEX2, MYH14, IFITM3, CC2D2A, DNMT3L, DSC3, WDR11, TBX4, BTN1A1, B3GNT3, NT5C3A, LHCGR, GPR179, CNR2, CD59, PLCG2, HRH3, TTR, CAPN13, HTR2B, CCR1, CRYGD, PGRMC1, MAOB, ITGA2B, CACNB4, PTGDS, PSAP, NCS1, CHM, CYP1A2, STX1A, LYN, BPIFA1, TGFB3, POMP, ELK1, HGD, BMPR2, KLF7, B3GALNT1, MYO3A, ADAM33, DNASE2, MGAT2, MSH5, TCN1, NOP56, PYGL, CTSG, GRIK1, CUL5, ICAM4, FPGS, NOD1, SOX5, KIF6, OTX2, DSCAM, SLC5A5, MCOLN1, IL12RB2, NFIX, SLX4, PLA2G5, PALB2, SEPSECS, RNASE3, IL9R, MED17, DIP2B, PIK3CG, BCORL1, PIGA, LPA, COL4A4, TPP1, IL17REL, C8B, UTS2, GJA3, TUBA8, TMPRSS5, BRAT1, STIL, NQO1, CYP3A7, RPL5, NODAL, HSPA9, FFAR4, AXIN2, HCCS, LHX3, MIR934, TAF1, MYO15A, S1PR1, CD79A, CIC, PIK3CA, HCN2, IRGM, ITIH6, SLC9A6, HTT, KDM4C, SLC6A5, DAZL, ERBB3, HAND1, CYP2C18, FGA, IGFBP1, IFI44L, SLC1A1, loc344967, FREM3, PSAT1, PTPN21, MOK, TBC1D1, IQGAP1, IFNG, TGM6, AGPAT2, EMD, RETN, APH1A, SLC6A19, SLC12A1, CYP27A1, GTF2IRD2, WDR35, FOXL2, HTR6, SLC7A9, SMC1B, KCNQ2, GRM5, PEX3, FGF2, C2orf71, CLU, PRODH, CXCL12, DIAPH1, CORO1A, OPRD1, C19orf12, COG5, AKAP9, LGI1, CRYBB1, SIL1, GYPB, FEN1, IGF1R, WDR72, UNC93A, SPTB, EFEMP2, DEFB126, PKD1L1, MIR191, PCM1, CAV1, ALDH1A1, NLGN1, FMO5, HPRT1, TNFRSF1B, RNASEL, DNAH9, FANCI, PIGN, IGHA1, SHROOM3, RAD54L, SNRPN, HMGCL, ALX4, CACNA1S, CORIN, ABCD1, PLEC, NCAPD2, ANK1, ASNS, POLR1D, GJD2, ABCB4, EPM2A, USB1, SETDB2, SFTPB, FAH, CARTPT, HNF4A, CNTNAP2, TNFRSF10B, USH1G, FRK, TBX19, CDK5R1, PAX8, OPN1LW, SNORD115-1, HIF1A, SLC2A4, TSEN34, RPS19, FEZF2, SLC26A9, KIF18A, C1QB, MAP2, TNFRSF11A, NOS3, TRAC, SEMA4G, CYP11B1, KCNIP4, CDC73, CFTR, VSX1, GSTM3, MIR96, SPRY3, KCNA5, WRN, SLC47A2, ST14, RHBDF2, XBP1, CEBPA, ACSF3, FBXW11, CRYGS, ALOX12B, TDGF1, ERCC6, NOS1AP, DGCR6, PRMT3, PTPRO, CD3G, CDKN1C, PPAT, SIRT3, AQP1, COL17A1, GFAP, NIPA1, IL7R, ACTB, LCE5A, TLR4, BSCL2, CFHR2, PCDH9, MKRN3, ADAMTS13, SLC30A5, HSFY1, ACVR2B, IYD, SUFU, NKAIN2, SPATA21, CYP2C8, MYO7A, CD96, ACVR1B, MIR17, ROM1, CSNK2A3, SFTPA2, MLH3, CD1E, IFNGR1, MAGEL2, SLC29A2, CD320, SMG6, MPP3, INSR, ARHGEF6, TGFBR2, TLR9, DEAF1, GLRB, AMN, ITGA11, PTCHD3, MBD3, MDN1, HLA-DRB1, PGC, FGF3, TNFAIP2, FOXN1, IER3IP1, PEMT, ARHGAP6, MCM3AP, KCTD7, NOG, MYO1F, ST3GAL3, HLA-B, PPP2R2B, TCF7L2, TCTN2, VSX2, GPD2, RBM8A, ITGA9, SCUBE2, VANGL1, DAOA, PIGO, FABP4, GPANK1, DDX58, AKT1, GBA3, MCM6, ABCA7, FRZB, RPGR, COL6A3, RP1L1, APOBEC3G, CATSPER2, PTCD1, GNPAT, ZFHX3, SCRIB, AURKC, CYP2B6, CAMKK2, FZD6, PITX3, PON2, UNC119, KLK1, MLXIPL, MAP2K1, GH1, HPS5, ANK3, SLFN5, KRT5, GNAT2, TIMP2, SIX1, CDH12, ARSE, SREK1, GTF2IRD1, CCDC22, MAK, BSND, COL6A1, OAZ1, COX4I1, AMT, SGCE, WNT4, PRSS8, KCNK18, FRMD7, TCF7, TDO2, SAMD9, KITLG, HSD11B1, KRT6B, SUMO4, SATL1, AKR1C3, SETX, CACNA2D4, DDR2, COL5A1, TMEM2, IL20RB, ZNF592, SERPINC1, CFP, DCTD, F3, CTSZ, DOCK3, STX16, NR1I2, KCNJ5, LMNA, CASQ2, APOA1, PYGM, PRICKLE1, WNT5B, TAS2R38, CRYBB2, SLC25A38, FTCD, PSEN1, CTHRC1, GPC6, HIP1, MCF2L2, WNK1, CDKN2A, LPAR1, TSHB, SCN2B, RELN, RECQL4, NKX3-2, HAS1, DPAGT1, ST7, TRPS1, CASP2, ATCAY, TM4SF19, DOK7, ATG16L1, LCE3C, JUN, CSF1R, MYL2, PTH1R, NOTCH3, NF2, ADAMTSL3, TREX1, TNFSF14, PRRT2, AOAH, CA6, ESPN, CEP57, AR, CRKL, SESN2, NUP62, GATM, AP4E1, ZFP57, MYO18B, PGD, RSPH9, ARHGEF10, MPG, GAD1, CASP12, AMELY, CLCF1, MIR30C1, DHX36, HDC, KLHL3, SYTL3, SCGB3A2, TNR, ADCY9, DPP10, ATP6V1B1, NOTCH1, PTPN13, SLC22A9, OCA2, SSTR5, PNPO, PDYN, DAPK1, SUOX, IDH3B, CDH5, NPC1, GSTT1, UGT1A3, CD40LG, CES1, TRAF3IP1, UGT1A5, SMARCE1, VRK1, B3GAT3, RASSF1, ICAM5, AARS, CYP3A4, SLC27A5, ACSM2B, IL7, SAR1B, SCN9A, APP, POU5F1B, GATA4, MNX1, C6, PRSS1, HLX, PTGIS, MDM4, ZNRF1, MAMLD1, NDN, HBA2, FBLN5, SNCB, TNNT1, RAB7A, PMS2, HELQ, ITPR3, CBR1, RNLS, ZNHIT6, SRY, HMSD, RAB2A, ACTN4, HPSE2, PLCE1, INSL3, MLC1, EIF2B5, KCNJ2, PINK1, PSTPIP1, CYP26A1, CNBP, SLC4A1, HSPB1, PMPCA, HSD17B1, DMP1, MPL, RMRP, CUBN, ADAM12, IGF1, SLC52A1, HSD17B10, HSD17B2, NUP155, RBM15, TAS2R43, FRAS1, DAZ3, GDF15, CYCS, TECTA, PRKRA, HMGCS2, B9D1, MITF, NKX2-5, GYS1, BTN2A1, DDX11, DNAI1, CACNA2D1, DEC1, SLC33A1, HRG, PROP1, PON3, RPA4, HCN1, SYCP3, MSH3, UGT2B17, FGFR1, FXYD6, MTNR1B, SPECC1L, CLCNKA, TTC37, HLA-DRB5, GOT1, GPR143, TMEM39A, PCSK1, HOXA1, PIKFYVE, UGT1A1, SYNGAP1, HOXA13, PCOLCE, FRY, CSF3R, UTF1, DKK2, IRX5, HERC2, HSD11B2, LRFN5, HNMT, ITM2B, PAPSS2, ASPRV1, CYP2C19, PIK3R5, UTRN, CDK11A, COL9A2, KRT1, PALLD, IMPDH2, TLR1, TENM4, AGBL4, ZIC2, SMC3, GSS, SIX5, FIGLA, CASC16, KCNA1, HSPA1L, SSTR2, CLN5, BIRC5, CHRNA5, CES2, GNAS, DDB2, DDX53, GHR, DUX4, DYSF, GOLGA3, CDKL3, MTRR, BBS7, HTR7, GLE1, RIPK4, ASB10, MS4A2, BHMT, NPC2, LAMTOR2, AVPR1B, MSMB, ALDH18A1, TPI1, DNAJC5, GATA6, RPS4Y2, MSH6, ALDH2, AIRE, C3, QDPR, MRAP, GJB1, PIN1, SLCO2A1, FOXA2, RIN2, PDE6C, SIPA1, APOC4, GSDMA, MIR34B, NDUFA8, UBR3, DNM1, UGT2B10, UGT1A10, SORT1, IGHG2, USP7, ASCC1, RBFOX1, SERPING1, KIR2DL4, MEIS2, FZD9, HESX1, TNFRSF10A, ASIP, SCP2, KL, GNAT1, IFT140, ALCAM, MPP4, ERLIN2, SLC22A6, CEACAM16, GPD1, ZNF41, CDH15, NPM1, BCAM, ADAM19, FKRP, HLA-DRA, SSX7, GCK, DNMT3B, GUCY2C, ADRA2A, TERC, DAO, ADAMTS16, WDR19, EDNRA, NIPBL, POGZ, EFHC1, GHRL, GUCA1B, SLC17A3, ZNF24, WT1-AS, FRA10AC1, LDHB, CRYBA4, HVCN1, RYR1, PENK, LTBP1, TRIP12, SMAD9, FGF8, KCNE3, LRRK2, MBD5, LIF, OXTR, ACTC1, KDM5A, PSMC3IP, EPX, WNT3, ATF1, ZBTB18, DYNC1H1, FGFR3, IL5, HES7, CLSTN2, KRT37, FSCB, BBS1, OPRK1, CDC6, CST3, SULT1C2, CHI3L1, WDR62, DHX16, PKHD1, HPS4, CRP, OCRL, TTLL11, GLA, NEBL, HSPA8, CEL, IMMT, PICALM, AGGF1, ADAMTS2, ARPC3, MYH13, NPHS1, TRPV5, COLEC11, HOXD13, TCF4, APOBEC3B, CDY1, MED12, ABCC11, GALNT18, MYH2, CYP7A1, ASAH2, AURKA, SRD5A2, PNPLA6, SPG11, KCNJ13, AP3B1, AKR7A3, IGLL1, DNAAF1, MIR182, CSRP3, FN1, MTR, MMP20, KRT17, DHFR, ZNF711, CHRNA2, TTC21B, TPMT, DGKD, LOXL1, IQSEC2, UBQLN2, NR1I3, MEGF10, PRRX1, FAM83H, KLHL9, GAA, SCN1A, STRC, RPL10, GDF9, ECE1, HCK, LIPI, CDHR1, CSTA, DISC1, GABBR1, MIR890, NIPAL4, SCN3A, CLTCL1, NUAK1, ALG6, CDSN, NPPA, LAMB3, MOGS, CATSPER4, OPN1MW, FMN1, CARD14, CCL26, RPE65, NCOA3, MTHFD1, PLA2G6, ZFPM2, DEFB4A, SEC63, PIEZO2, FTH1, GC, PAFAH1B3, ADRB1, ALDH6A1, SNTG2, G6PC2, MUC5B, CTSB, ZFP36, UMPS, TNF, COG4, FANCA, BFSP2, PGAM1, FRMPD4, GRM6, IKBKG, NGFR, KLHL10, MEIS1, NSD1, CRYBB3, EDAR, NEDD4L, EGF, GNRH1, IL1A, BRCC3, CACNA1H, ERCC1, CAV3, HARS, KCND3, AGO2, TRIL, CREBBP, AKR1C4, PARK7, PAX1, AP4M1, COQ5, TUBA1A, SGK1, ABCC2, COL5A2, NOP10, SELP, IFT43, NAIP, MYOZ2, XRCC4, SOX10, AOC1, KIT, SIGLEC16, TRAPPC2, KRT8, PDE6H, EPHA7, SCN4B, PPOX, SERTAD1, HAND2, NSMF, ANTXR1, RHCE, DGAT1, TAC3, RAD54B, ATG7, MED25, SPP1, GLI1, FBN3, BARD1, DGCR5, SFTPD, MCFD2, MIP, F2, ZNF627, EIF2B2, WNT5A, TRPA1, GNS, FDFT1, PEX26, PTGS2, BMP7, CFHR1, ADAMTSL4, LRP5, ARFGEF2, KLK4, NEFL, CTNNB1, GALNT2, NAV2, ADH1C, DND1, ATXN7, EFCAB5, STK3, IL21R, REPS2, ANO10, FCGR3A, CYP2C9, DNMT3A, FSHR, FCAR, WNK4, KRT83, BMP15, GAD2, ITGA6, BAG3, C1GALT1, CCND1, CTSK, PIK3C2G, PRKDC, IMPA2, MSR1, TRPM2, EXOSC3, ZFP90, ATXN2, RS1, S100B, BMP2, LAMC3, NPY1R, UGT2A3, PCDH15, CHDH, ESR1, GCNT2, GABRG3, SLCO1B1, SST, KCNN3, PRF1, CDK4, GIGYF2, ITIH3, CD79B, EDA2R, IRS2, CLN6, ANAPC1, DMXL1, PTPN14, GPR1, HTR5A, GRM1, RAPSN, PDE4D, TRPC4, ROPN1L, GEMIN4, GAP43, KCNMB3, KLK12, PCDH11X, TMC6, DMGDH, RIMS1, OTOF, XYLT2, TEX14, RRH, DRP2, DHCR7, PDZD7, FGFR4, SLC19A1, POR, SREBF2, PIK3CD, ACAN, TGFB1, ALG11, DOCK4, APEX1, ZIC3, CEP68, EOMES, PAX2, SCARF2, MPDU1, PSEN2, KRT31, PTGIR, FOXE3, ART4, PRM1, NLGN3, DAG1, WWOX, TIMP1, ABCA12, GP2, PLOD2, CHIT1, RPS24, PCSK2, PI3, MRC1, MAVS, SMG1, IL13, CD86, FAM91A1, LFNG, PIP5K1C, CHD1L, GPT, CNKSR2, CSAG1, FUT8, IBSP, AGA, SNURF, B9D2, STAT1, CPZ, POLR2M, IGFBP5, ATXN3L, NRTN, MMP12, TNFRSF25, PEX11B, GABRA1, HABP2, REEP1, BMP10, MYOCD, SLC2A1, CER1, ATP2A3, PTGER4, CCR7, KCNQ1, EPCAM, BCL2A1, CTTNBP2, ZBTB24, SPAST, PLXND1, NLRX1, BSG, SLC26A6, PDE7B, FN3K, MS4A3, EFEMP1, BIN1, FGFBP1, ALS2, TRIP11, MOCS1, DOCK6, SYP, GLCCI1, PTPRC, OLFM2, MYO1E, DUSP23, CD14, ABCC9, SNAP29, HMGCR, WDR45B, CDH3, INSL6, NR3C2, NPAS2, PRSS3P2, IQGAP3, CRADD, BLNK, CHGB, COL9A3, IHH, IL19, DIAPH3, DOC2A, XKRY, UBR1, GGH, ADARB1, COL9A1, MCCC1, VPS35, GPC4, COL4A2, SRCAP, LUM, C1S, BPGM, TCF7L1, GFPT1, HMOX1, GLO1, MC4R, FAM126A, TRPM7, INS, ERAP2, FLT1, PFAS, ALOXE3, EXT1, SMAD2, FCGRT, EIF2B4, SIX6, CCR6, TSSC4, TSPAN12, COL11A2, PLD2, ASL, GATA3, TFAM, ZNF365, ALG10B, ARX, LDLRAP1, ANGPTL4, HHEX, GTF2H1, FLCN, MPZ, NAMPT, CYP4F22, ABCA10, UROC1, FOXC2, HDAC4, GLRA1, CTNS, GDNF, FAAH2, TRPC5, SCGB1A1, TEP1, GSTM1, CRH, ACP5, TBCE, CA4, PLCB4, HSD17B3, SAG, CTRC, HOXA2, TSC1, MSH4, DKK3, TULP1, CYP27B1, FTO, VPREB1, USP46, SPI1, GYS2, PLAT, TNFSF11, RPGRIP1L, PTPRF, BLVRA, POLL, FRG1, TYK2, MUC6, MYL3, NRL, THBS4, SDHAF2, RAX2, PIP4K2A, MYLK, CYP4F2, UNC5C, HOXD10, CXCR3, KIF17, CHI3L2, GPBAR1, SRPX2, CBR3, CTLA4, UBAC2, PLA2G2D, AQP3, RAB40AL, GLMN, KRT6C, CSGALNACT1, APOH, PDX1, LCN10, PRPF31, TLR7, SMAD1, PLCD1, NPHS2, RAB3GAP2, CLCN1, CLEC7A, SOGA3, IL17F, MUT, IL4, SELPLG, NAT2, TTC8, POU6F2, OPCML, ALDH16A1, F13B, KRT38, HLA-DPB1, XIST, HSD3B2, GNE, MGAT4C, CEP85L, SLC39A13, ARL6IP5, TUSC3, GABRD, LIPG, MYF6, MGST2, TRPV4, APOD, NEK8, SULT1A3, RBBP8, HDAC8, PLCZ1, MED13, SIGLEC12, SCN1B, ACVR1, DNAAF2, STAR, RNASEH2B, F2R, PHF2, KLB, TAS1R2, MTAP, ADORA3, COL6A5, ASMTL, PIK3R2, GZMB, FCGR3B, BMP1, CYP3A43, AKR1B1, SRD5A3, HLA-G, TMEM114, CYBB, LRP2, SART1, SPTA1, GEMIN2, SEMA6D, PMAIP1, SLC25A20, KCNJ3, MYCN, C2, RPS6KL1, NTF3, GCKR, TRPC6, GSPT1, SNRK, ANKK1, GUSB, GSDMB, EXTL3, LIG1, HYLS1, DNM2, EHMT1, GLDC, FBP1, PLA2G7, RAX, TERT, PDE6B, DLGAP3, FUCA1, KRT18, SOCS1, CDK5RAP3, IL17RA, YTHDF2, ST5, GGCX, STRA6, CAMK4, ROBO3, CPLX2, RB1, PHLDA2, TST, SLC35D1, NDUFA7, POP1, SLC29A1, SAA1, ALG8, SHOC2, SRC, FBXW7, NTNG1, CYB5R3, NR0B2, TFAP2A, ADTRP, PDE4B, ZNF507, IL1RN, MAFB, TCTE1, RFX6, PHF8, ADIPOR1, DNAJB2, TFPI, FABP6, SGCD, BBS5, CYP17A1, TWIST2, SCG3, ESCO2, CD209, GJC2, SBDS, ENO1, GABRB3, PCBD1, COL6A4P2, GIPC3, MAN1B1, TPTE, GOSR2, BFSP1, COL4A1, STK19, GRM7, TNFRSF13B, GLS, STS, LGALS13, IL4R, LHFPL5, GLI2, SHROOM4, CCL5, BTNL2, TGIF1, TP53, PEX5, ENTPD1, STK11, MX1, LOX, YARS, EME1, MIR184, NLRP1, UNC80, LAMB2, HNF1B, TGFBR3, UMOD, CASP9, PDCD1, ATP6V0A1, IMMP2L, MIPOL1, ZNF469, LAMP2, CAT, SLC7A1, ATXN1, CPOX, LAMC2, TIRAP, KIRREL3, APH1B, ADAM17, CTNND1, GABRG2, CHRNA3, F5, LYZ, TPM2, CPS1, CNDP1, ABCA2, ATP10D, CFLAR, IGBP1, PRKCH, NRXN3, CDKN2C, ACACB, STXBP1, ADM, PREPL, FGD3, RHPN2, GHRHR, ABCB6, CD22, FCGR1A, IMPDH1, PRKACA, PPP1R1A, RPL11, KATNAL2, TRPV3, NMU, ASXL1, IFIH1, IDH1, SEMA4A, XRCC5, AHSG, POU5F1, INHA, PMS2P3, CLDN1, ICOS, NFKB1, SIRT1, MANBA, ABCC1, GFRA1, NKX2-6, RBP3, PRM2, IL1B, MUC13, HAX1, REN, GDF1, ACTA1, WRAP53, TDRD7, SP8, CTSA, CDKL5, KCND2, NSUN7, ELP2, WIPF1, PIP5K1B, AGXT, IGF2R, INPP4A, MMADHC, DAZ2, ATP8A2, IL12B, SIAE, ZMPSTE24, DDX39B, UBE3A, MT1A, SNRNP200, DDX25, NCAN, SOBP, ASMT, RNF114, ORC4, ITGA7, TSSK2, TXN2, CLEC11A, DNAL1, TAF15, HAMP, SREBF1, IDE, NOD2, CERS6, GPR68, IL21, HTR2A, LTK, FUT7, SCNN1G, EPAS1, RAB18, COL8A2, GYG1, TPH2, PHB, LPIN1, KRT12, BAALC, TNP1, WDR13, HSPB7, PMP22, PGAM2, PEX12, APC, CNNM2, LOXHD1, TLR2, HTR3E, GPR139, GPX1, SBNO1, P2RX7, LIMK1, POLR2E, PSCA, LRAT, HIGD2A, MACROD2, TBX6, IL18R1, BCL10, GLB1, GAS6, TSHR, MOCS2, HCRT, NAT1, SULT2B1, CYBRD1, MTCH2, PEAR1, AUTS2, MMP10, TCN2, CDA, TFF1, ZFY, PPARGC1B, EPHB2, ACTG1, CHRNG, CALM1, TAS2R50, PLAUR, NME1, BMP5, CKM, CEBPE, ESR2, AGTR2, BHLHE41, MESP2, DNAI2, PZP, RBM28, CD3E, APRT, MEP1B, TP53RK, KCNJ8, ITIH4, TCOF1, SLC16A1, GJA5, SLC17A5, HGF, SOX18, SERPINA10, INSIG1, SYNGR1, CFD, ACSL4, PPM1D, NHLRC1, SOX2, CYP1B1, COMT, CACNA1G, AMER1, PDLIM5, XYLT1, GNPTG, ROS1, BRWD1, ZFAT, TRADD, AS3MT, SLC6A3, KRT75, SLC19A2, CD3EAP, C9, PVR, BEAN1, FANCG, DCAF17, ZNF224, AVP, TMPRSS11A, L3MBTL1, SCN3B, PGK1, OAT, CCL13, DRD1, IQGAP2, PLOD1, ANKS1A, SMNDC1, RYR3, PRCP, XPNPEP2, SPINK1, RNF135, PDE6A, NBAS, NDUFB1, SLC5A11, DHCR24, ADRA2C, NBN, ADAMTS18, SLC17A1, CYBA, SOS1, PLAU, RAC1, KMT2E, PTEN, ARSF, C1R, SLC28A3, ATP1A3, PNKP, SETD2, ROGDI, MATR3, TICAM1, CILP, PROZ, BTLA, SERPINB5, SEC23B, DCK, RFX5, SLC6A4, SPTLC1, CALR3, TOPBP1, PKP1, NDUFA6, KLF11, SYNE2, SLC22A4, SERPINA6, GALK1, FABP7, DPYSL2, FBXL6, SDCCAG8, KEL, CDK5RAP2, FSCN2, FTHL17, AADACL2, NGF, GK, SLCO1B3, DSCR8, FLVCR1, PAX4, GIMAP8, CEP135, ALOX5, NR2F1, TRIM22, ACAD8, CDH13, NLRP3, GJA1, CCDC50, FASN, GNAI2, PHLPP2, RAB3GAP1, ASCC3, TGM2, ZNF202, POU3F4, DEFB1, GRM8, ARHGEF7, TBK1, PKD2, CRYM, NR1H3, SLC44A2, EPHA2, SQSTM1, AADAC, HPS6, PROK2, RPGRIP1, ACAT1, MTHFR, PTPN11, PHKA2, KDM5C, ATF3, TRDN, SNORD116-10, UCHL1, MPP7, POU1F1, NPHP1, FBN2, RTN4R, SYT2, PHF3, DNAAF3, GDF6, LCT, DICER1, APOC3, NPPB, CNOT4, IFNA2, PLA2G2A, FOLH1, SLC16A12, CASP10, ALK, GAN, PCMT1, HIST1H2AE, SRI, HLA-E, EVC2, TAF7L, YWHAE, ATP1B1, GON4L, NEUROG3, MGST3, BDNF, CDKN1B, LMBRD1, PNMT, LDLRAD4, SEMA3A, FOXC1, BTRC, STEAP3, EIF2B3, ACBD6, HSPB3, ABCA1, FGG, SEMA3E, CSF1, PQBP1, TNFSF10, SMARCB1, MUC1, TAS1R3, MYO5B, C3AR1, ACOX1, USP24, KIF5B, MUC7, CSDE1, MYH9, CLPTM1, LBP, BDKRB2, HSD17B4, AICDA, DCLK1, TLR6, MEF2A, GPI, RAD9A, HTN3, TAF2, SLC4A3, ADORA2A, CDKN2B-AS1, STIM1, KCNH2, TBX2, FOXD3, TMEM237, IFITM5, CCHCR1, RPS3, RPS27A, ADCY5, GCM2, FKBP14, TNNI3, SGCB, IGFALS, NPAT, NR0B1, DUOX2, RASGRP2, PRKD3, CASP8, APOB, PIGR, CFH, TSC2, NRIP1, NRP2, FUT6, PTPRB, GJA8, FGF20, FLNC, KRT3, MOG, KRAS, SPTAN1, ERAP1, ATP7A, ITSN2, GIP, PSPN, C4A, HOXB13, PPARG, DSG2, BBS10, GYPE, SIGLEC14, PPP2R1B, APLNR, JAG1, CHGA, IRF6, MMAB, EVI5, CALCA, HTR3C, IFT80, ENAM, CXCL11, DLL1, TNNC1, AKR1C2, GNB1L, IL31RA, MYBL2, GFI1B, PPIG, C12orf10, MAPT, NAGA, MRE11A, HLA-DPB2, HOXD4, TTBK2, C8orf37, GJB3, HLA-DQB1, NRAS, MTTP, ECM1, SIX3, CHRNB4, OGG1, NYX, HDAC9, EPB41L1, DYM, LITAF, TMEM165, AFP, MSX2, PHOX2B, MYT1, CPB2, CTF1, USP9X, TGM5, SLC34A1, NR3C1, FCN2, DLX6, NPAP1, F11, ATP2A1, ZC3HAV1, YY1, ZNF80, SLBP, MIR124-1, FBXW4, NBPF1, WT1, TAS2R46, CCDC170, STEAP4, TXNRD2, HTR3B, TNFRSF4, CBLB, MIIP, SNAPC4, SLC22A11, HMX2, VAPB, IGHM, TAS2R19, FBLN1, GSTO2, MRRF, RP1, RAF1, INF2, ZIC1, PRPF6, KLRK1, LYST, FURIN, ADH7, P2RX5, NCR3, FKBPL, APOA5, CCR2, EDN3, BLM, GMIP, RIMS3, DRD2, UNC13D, SMAD7, SOX3, CFHR4, GATAD1, TMC8, FCER1A, FHL2, CALR, NUDT6, PHOX2A, KCNMB1, DES, S100A14, ASPA, FGFR2, XRCC3, GDAP1, GH2, BEST1, MMACHC, REV3L, PTK7, RBM20, PHKA1, PRKAR1A, HSD3B1, TNNI2, RAD52, RGS7, CD207, DISP1, RSPO1, FEM1A, CHUK, HR, PDPK1, GM2A, CPT1B, KYNU, SRGAP2, STK32A, PCDHB4, CD2AP, SMN2, PEX7, RPS26, STX11, CYP7B1, KCNC3, TBXAS1, STXBP5, HSF4, LOR, ARG1, KIF5A, PSENEN, OPTC, SPRED1, PCK1, HBD, NPR3, TRBV9, RAD51C, CNNM4, CFC1, IRAK3, MYO5A, ADAM10, SULT2A1, PRPS1, GALC, LIPA, TMC1, CNTNAP4, CHST14, PPP2R2C, P2RY12, PNPLA1, SLC6A13, KANSL1, VWF, LHX8, MCHR1, VTN, TPM1, TBX1, CIDEC, ZNF644, MPHOSPH8, CYP2E1, FLNB, WDFY4, OPRL1, CTH, ATF5, SPTBN5, TAF1L, STK33, SLCO1A2, LIG4, ECSIT, BMP4, VSIG4, OSTM1, CLCN7, AGTR1, MC1R, RNF168, OBSCN, CISD2, CLCA2, GBA, SLC52A3, DPY19L2, CYP2G1P, PPIA, CSNK1D, CCL4L1, NDOR1, TRIB1, PRKAG3, NT5C1B, KIR3DL1, TPRN, CD46, RALGDS, PTPRT, PER2, RNU4ATAC, RGR, CCKBR, DSG1, CCK, NDUFB6, ADRB2, CD3D, TBP, RPH3AL, CSMD1, CTSD, HPGD, NHEJ1, TRERF1, TUBB3, SLC24A2, IL3, MIR510, ANO7, GLI3, TP53BP1, PTCH1, ERCC3, COL25A1, AMACR, TFRC, TINF2, SLC7A10, AK2, VPS13A, CYP4A11, BCOR, CRYGB, AVPR2, GRIA3, CRISP2, RP2, NTRK3, SERPINF2, FMO1, PICK1, MIAT, SGCG, FBN1, DCDC2, C12orf57, FANCF, BEX4, TBL1Y, HYDIN, SLC10A2, PEX6, TMEM187, AIP, MCEE, TNFRSF13C, SLC6A14, MAP3K1, ALDOA, CTNNA3, GAB2, AP3B2, IQCB1, LAMA5, HNRNPH3, SKIV2L, DIS3L2, FREM2, TBX15, IL12A, PRB3, FUZ, DROSHA, BMPER, CCL2, RGS9BP, KPNA1, MIR125A, KRT81, RFXANK, SPATA7, ADA, LAMC1, EFHC2, PDCD10, EIF2AK3, CHMP4B, ACADS, MS4A6E, TROAP, EPHX2, ANXA5, ELOVL4, KRTAP1-1, ANGPT1, KARS, MED23, PTGDR2, CCDC8, VIPR2, PRLHR, HMGA2, EIF4G1, TMPRSS15, TRPM4, GJC3, ISYNA1, RAB39B, HSP90AA1, ICK, SEL1L, NDUFC2, ATP6V0A4, SLC4A10, PARP1, PLIN1, FGFRL1, BAG6, LHX1, KRT2, RPS15, ITGA4, SEC23A, DNAJC6, CLOCK, DSG4, CPA6, NSUN2, JAG2, ROCK1, TGM1, SLC37A4, PTPRK, WDR81, AP5Z1, PRKCSH, ENG, ARHGAP31, KLF1, RXRG, SNORD116-1, SULT1E1, KDR, SORCS1, TBC1D4, GLUD1, GIT1, HPD, RPIA, PHIP, MYLIP, SLC11A2, NBEAL2, EPB41, TYROBP, MEN1, RMI1, IKBIP, ECM2, NPHP4, TNC, MYD88, DOCK9, PML, BRAP, CBFB, EBAG9, UROD, RPL36, ASPM, LRP6, SERPINH1, AFF2, RFC2, ZDHHC8, PPARA, CD244, ZDHHC15, KRIT1, LEFTY2, MGLL, RORA, PTPRD, IL2RA, MYH15, CD24, CRX, MIR892B, KCNJ1, PRG4, ARHGAP9, UGT1A9, CRYAA, ATP1A2, BACE1, TNFSF4, TG, OPTN, ACADSB, BCL2L11, CABP4, HDX, PRSS12, SLC28A2, RBP4, SP100, IFNA17, MYO1A, BTC, PLCB1, RAI1, LAMB1, LOXL2, IGF2, PROK1, SERPINA7, CHRNA1, RPL21, PHF6, HOMER2, DNMT1, HTR1B, KCNS1, BRIP1, TRIM21, SBF2, CFI, ADRA2B, KIR2DL3, SLC7A5, FABP1, T, SORBS1, SLC15A1, NPC1L1, TAT, GSPT2, CRYGEP, LIFR, CBL, GSTT2B, GP9, DMBT1, KCNAB2, GSTK1, SPTLC2, ZDHHC9, CLCN5, TSEN2, SGCA, MAGI2, NLRP2, FLT3, NELL1, PCDH19, TSPO, ADORA1, HES6, UCP1, POLE2, FADD, FGF9, FOXA1, IL2, VNN1, CACNA1A, SPG21, CDH1, OSR1, GRIN1, ITPA, EMC4, EDA, CHRNA4, HSPA5, SERPINB6, TNFSF13B, CMPK1, SCN2A, WNT7A, UPK3A, FIP1L1, SSH1, RHOB, ENSA, H6PD, GNAQ, XPA, MMAA, PRB1, APOBEC3H, AMH, ZNF526, GNPTAB, IRF5, ACAT2, GNMT, HADH, TRPM1, PPP2R1A, CCKAR, TMEM173, COL2A1, ARL14EP, RPA1, TROVE2, IFI30, PLEKHM1, GLUL, MSX1, WNT10A, HK2, RDH12, MICB, EPHX1, SLC30A10, DLX3, AIMP1, AP4S1, SLC27A4, AVPR1A, GJB2, SOX8, ATXN8OS, ICAM1, MTHFS, ZNF674, BRSK2, CD19, SCARB2, SLC20A2, CRBN, STK4, SLC16A3, DLX5, ADK, CELSR1, TNNI3K, CHRND, THBS2, NEXN, CA2, FOXF1, TPO, LTF, TTI2, TAS1R1, ETS1, FGD1, AGRP, HSP90B1, SMOC1, TMEM138, GSTA3, HOXB6, USP26, CD81, MYH7, TCIRG1, SH2B3, CYP2W1, TMCO1, PARL, BICC1, TRRAP, RYR2, GRN, TRIM32, HOXA4, PTS, ADCY6, SLC36A2, DDC, NXF3, NPR2, PROM1, PRLR, ZFYVE26, GDF5, SNX3, CHIA, CATSPER3, TBX20, INMT, ADSL, FTSJ1, DST, BAP1, NPSR1, GLRX5, MSH2, LNX2, NPPC, ZEB2, CYLD, HIF1AN, ABCG5, BUB1B, MIR16-1, USF1, JMJD1C, SLC46A1, MTMR2, CHRM2, PRRC2A, NR1H4, RRM1, COX7A1, SALL4, PDE11A, CADM1, NOTCH4, ZCCHC8, CD5, ABCA4, OPRM1, MAT1A, CLYBL, CYP26B1, CALM3, APCDD1, RGS9, PCCA, CNKSR1, B4GALT7, AQP5, TSSK4, CEP41, SDHC, MPDZ, ATF6, GNB5, EYS, FMO2, IL16, ITIH1, MSTN, VCL, RNASEH2A, SERPINE1, CCDC88C, HFE, SMN1, BRWD3, VCAM1, EDARADD, PTAFR, PORCN, ROBO2, ALG3, CR2, GNA14, LPAR6, ZMYND11, MYH8, CDH23, PEX10, TTN, SLC26A5, RGS6, GALE, SRGAP3, NCALD, LMTK3, AKAP13, FLG, SLITRK1, ALS2CL, HHIP, RAD51, RAD51D, ADH1B, MEST, FERMT1, DYRK1A, HTRA2, SARDH, RANGRF, HBS1L, WISP3, CRK, KIAA0319, RNASET2, PKM, NEDD9, RPS7, JAK3, HSPA1B, FCER2, CXCL5, SPRR3, GSTO1, PITPNM3, ENPP1, SCNN1A, IDUA, KCNE2, P2RX4, CFB, UBIAD1, CDC42BPB, FCN3, SLC34A2, NPTN, NPY2R, SART3, CAPN10, FOXP1, CCR5, NDUFS5, SLC5A1, EPHB6, SYT14, PDLIM3, SCN8A, UNC93B1, RGMA, TJP2, KRT10, LILRA3, CCT5, RAB27A, CSF2RA, PEX19, PAX3, EYA4, RALGAPA1, YAP1, EDN2, PSMD7, ITPKC, ST8SIA2, RAD21, PPP1R3C, LMX1B, LRP4, SLC5A2, DLGAP2, MIF, PFN1, ORC6, IRF8, POMT2, CHRNA9, ITGB3, SPATA13, CEP152, CFHR3, BMPR1B, PDE8B, SF3B4, MAP7D3, LTC4S, SMARCA2, TUBGCP6, TLL1, ADAMTS10, COL10A1, BLOC1S6, NLGN4Y, BCL11A, F12, KDM5D, ACTA2, FANCE, FZD1, MKL1, ANKRD1, FREM1, SAA2, DDR1, PTGS1, ALDOB, LAMA4, DCC, FBXO18, TMEM185A, SYNPO, GUCA1A, UBE3C, CLIC2, KLF10, ASCL1, LTA, TTPA, IFNA10, MIR499A, MGEA5, UBR7, PVT1, CYP46A1, BCAT1, PARD3B, SLC2A9, CHN1, RAG2, HGSNAT, PMM2, HMGA1, KRT85, SLC14A1, CA1, RANBP2, DCP1B, LIG3, SERPINI2, IRAK4, KCNJ15, ECI1, DHODH, TULP3, ATP2A2, MLPH, ATIC, CXCL16, ATOH7, NRG1, NPL, CAST, TGFBI, SLC4A11, SNAI2, DGCR2, MARVELD2, CISH, JPH3, SPINT2, CYP2J2, CASK, HSPG2, APOL3, FLNA, BAX, CP, LRPAP1, SLC17A8, G6PC3, CYP24A1, SMARCAL1, ANO6, NPAS3, PRMT7, AHSP, USH1C, DNAH5, CTC1, GHSR, ATP8B1, CLN3, TRPC3, SEMA4C, FMN2, TUBB1, SCN5A, FANCB, KAT6B, FBXO10, PDGFC, TP63, PEPD, ANXA11, VHL, CSF2RB, LMBR1, ZPBP, RPS6KA3, FGF23, NLRP14, PNPLA2, PCNT, BCKDHB, GALNTL5, PAH, PTCH2, ERCC2, VPS13B, DCLRE1C, HBG1, PSMB9, CYP2R1, HCLS1, CSF2, GHRH, IL11RA, RPS6KB1, GDF3, MMP9, POLR3B, RXFP2, HBA1, CTSC, AQP7, ADIPOQ, MPST* |
| Mitochondrial genes*  AND  TruSight expanded† | 33 | *PNPT1, SUGCT, ATP5A1, ATP5G3, TARS2, SFXN4, LYRM7, FBXL4, RMND1, UQCRC2, SLC25A1, COA6, COX20, VARS2, COQ8A, SERAC1, COQ8B, LYRM4, CLPP, TRAP1, OGDH, MICU1, COX7B, CA5A, DNA2, IBA57, APOPT1, MPC1, CYC1, MRPL44, MGME1, IARS2, SLC52A2* |
| TruSight expanded†  (not overlapping) | 1933 | *DPM2, FSTL1, CREB3L1, RIT2, SLMAP, FEZF1, SAMD4A, ITGA5, APBB2, ECHS1, NAPRT, PBX4, LRRC41, HOMEZ, DDB1, C12orf66, KCNA6, ATP5G2, ERG, FAT4, PARN, LRMDA, KCNK3, PDE1C, SEMA4D, MREG, HEPH, AK7, PIR, IGSF3, LONP1, ARL6IP1, CHD1, C15orf41, WDR34, GLRA4, OR10X1, KIAA1210, GEN1, UNC45B, BMP3, SLK, BAZ1B, CCDC66, PPP1R15B, LRGUK, SLC25A46, LARP7, ELOA3D, RNF32, HES1, RAB33B, CIAO1, KCNC1, C2CD3, TNFRSF9, CCNE1, FOXM1, FBXO31, FBXO11, ST8SIA4, CDK8, EMC1, ORM1, POLR2B, B3GNT7, RPS28, TUBB, PLXNA1, RFX3, CRELD2, CD82, VAX2, SETDB1, KLHL41, GMPPA, OR4M2, HPCA, MLF1, FCN1, CLCN4, RNF39, DNAL4, IPP, POLR2J, UQCRC1, ZBTB11, CEP120, ADAM23, B3GALT5, SPTBN1, RICTOR, GRXCR2, RHOH, GNA11, ARSH, TACR1, CAPN1, DDHD2, UNC13B, MYO5C, SARS, SLX1A, LAMB4, PRDX4, KRT71, DAPP1, CNTN2, CAPN12, ECEL1, EPHA4, KCNQ5, C21orf59, ZBTB20, SELENOP, WASHC5, CENPX, LTBR, GNB4, ANKLE2, SLC9A3, AGTPBP1, SLC39A5, FKBP4, HCFC1, KIF14, SCN11A, SMC6, RTN4RL1, EIF4A3, DLEC1, KCNA4, ARID5A, UBE2N, IGSF8, ACAD11, LMO4, TRAF7, HUS1, DNAJC30, HID1, POLD2, RTEL1, FSTL5, PDAP1, BRD4, ARFRP1, KLHL6, CASC4, PCDHGB4, PDGFRB, DTNB, rs6841061, SLC4A5, HOXB8, ITSN1, SVIL, PHB2, TNKS2, DUX4L3, NDUFB8, CADPS2, MYF5, GRHL1, DLST, FST, ZNF419, MTMR12, ABCC12, FAM177A1, GSDME, TANC2, KIF4A, TFE3, OVCH2, TMEM189, LGR5, DCAF11, LRP1B, SULT1C3, TLX1, OR2M2, BDP1, RPS29, OR8K3, KMT5A, TRIM63, PLRG1, BCL7A, PARD6G, INPP5B, FAM71A, CLIP2, ERMARD, PARP4, NCK2, OXA1L, SC5D, CEP164, E2F4, TAL2, HPCAL4, NFAT5, NUB1, rs8045964, DVL1, FLI1, MAB21L2, MELK, PODXL, CLIC5, HBQ1, ARHGAP26, CDK16, JAGN1, KRTAP4-8, SPATA32, MAP3K6, SDR39U1, PPP6R2, PGBD3, DMBX1, AREG, LIPT1, AP2S1, KANK4, ZNF713, SGO1, SGSM3, NR4A3, DNAAF5, MUS81, AGBL1, OR1B1, CHPF2, FGF22, C21orf2, RPL27, NLRP9, NIPA2, MAGI1, BGN, PJVK, RDH11, PIGT, FOXO1, KIF5C, MATN4, SMIM1, PPP1R17, PIAS1, CDH20, ST6GALNAC3, PPP4R1, ZMYND10, WNT16, STYXL1, SLIT1, DMAC2, ADCY1, TCTN3, TRIM24, PLXNA4, GBA2, SYN2, KIF23, SEPT7, ADGRG1, ZDHHC17, KCNH1, HFM1, SPEG, PTPN23, HIST1H4B, FAM3C, KCNH3, AP1S3, SLC12A7, MDC1, PRKN, PYCR3, ATP11B, TELO2, RINT1, NRN1, DNAH7, DUSP3, RAD23A, IFT172, CTNNA1, SCAMP4, NDUFB7, DCAF4, SPANXN1, OR8H1, ACR, RAD9B, PADI4, AK8, BIVM, NDST3, KLHL15, GALNT6, SNX5, RASGEF1B, NCAM2, PACS1, NR4A1, PURA, RPL15, TRAPPC11, NEO1, MELTF, RASA2, STAG1, NECTIN4, DHX38, UFD1, CFAP53, CLPTM1L, EML4, ATP6V0C, NOX3, ARHGEF4, C2orf80, SZT2, ARHGAP29, FBLN2, PPP2R2A, SCLT1, CAMSAP2, ABL1, ZNF433, LY6G6F, MKL2, DAXX, SLC24A4, rs10748087, GAL3ST4, WDR27, PKHD1L1, MCC, IFT88, P2RX2, NME5, CHMP1A, CCDC85C, MESP1, MTA1, BPIFA3, CLCN3, GNG7, POLR2A, MECOM, POLE, TSPEAR, CAMTA2, SCN7A, TRMT10A, NLRP8, MCF2, CKAP2L, POLR2J3, C16orf58, DDHD1, PPP6R3, BICD2, LRRIQ1, TCL1B, FGF13, PPP4R2, KCNV1, ACP1, ZMYM2, B3GNT6, KIAA1586, KIZ, TGM4, FAT1, MGAT3, SIM2, GTF2H2C, CELSR2, DOK5, PTRH2, ZNF668, STAMBP, CRYL1, NEXMIF, PPM1G, KCNC2, SNX8, CDH19, TAAR1, SELENON, HDAC1, ELMOD3, RNMT, PROX2, MYH4, FAM46C, DCHS1, SYCE2, CXCR6, TPPP, UST, UBE2V1, MBOAT7, ST6GAL1, ATP2B3, PYGO1, RAB10, ANO3, HIBADH, DEK, CRTC1, UPF2, LMO1, DNTT, SUCLG2, JMJD7-PLA2G4B, AMPD2, ACTN1, MRPL36, SPRED2, BBIP1, ELOB, POU3F3, KCTD3, GULOP, NKX2-2, LRIT3, C14orf2, KCNMB4, PYCR2, DOCK7, ABCA5, PDK3, GAGE8, BICRA, TNPO2, IRF9, LBH, MYO1D, UGT8, POMGNT2, HINT1, OR52N4, RPL38, PRCC, CHPF, RFC5, MAP7D1, IGKV1-5, CYR61, rs4411641, MYBBP1A, EHHADH, USP8, LAPTM4B, NEK2, WDR73, VWA3B, NDUFA5, UQCRH, PGBD4, ACKR3, MAEA, ISCA2, RDM1, ST6GALNAC1, HMGB1, EIF2D, CBFA2T3, RAP1GDS1, PLXNA3, CDC25A, FZR1, DPH1, MMS19, ESPL1, ST8SIA5, B3GNT5, USP30, MAP4, UGCG, STT3A, QARS, SOX7, ARV1, COX5A, MACF1, WWC2, SEC24D, POLR2L, EPB41L4A, MNAT1, ZDHHC2, TAF4B, MTA2, SLTM, RAP1A, MALT1, LZTR1, KANSL2, ZNF783, FCRL6, HOXC13, METTL23, SMARCC2, RECQL, NLRC4, POLI, SMARCC1, CLHC1, UCN, GNAI3, SPATA5L1, RAD51B, MMRN2, SOX4, DUSP22, SLX1B, C18orf8, ZNF347, ST6GALNAC5, GPNMB, TTF2, SFPQ, KCNT1, PTPN12, ABL2, CRYZL1, CRY2, PTPRZ1, GPR137B, TOP3B, RXRA, STAG2, MAML2, NFATC1, SSX2, INTU, EML1, CDK12, PLCG1, MEPE, SLC16A7, GALNT13, SPO11, NEDD4, DCBLD2, ZBTB38, CFAP57, SLC25A5, PI4KA, MRPL12, NLRP6, TIPARP, PGAP2, CHEK1, NEIL3, RTN3, ZNF175, MRAP2, MAP3K14, PPP1R8, PIBF1, PDE6D, TTLL1, PRKG2, CCSER1, PPP6R1, SPRTN, SUPT5H, SOS2, APOL4, LARS, ANKRD28, GRK3, GALNT5, PLXNB1, ALG14, ARHGAP45, STAT4, FAT2, rs6512586, ZNF311, PRMT2, ITGA1, ECE2, GTPBP3, TCF12, ACTRT1, SLC7A14, ITGA8, DCLRE1B, PRKCD, NDUFB4, SYNE4, rs6666954, SLC12A2, RIPK2, DNAH1, ZIC4, ZFP36L2, PPP4R3B, CST9, PCLO, ZNF778, USP54, RNF2, MTF1, SCNN1D, NOL3, NLRP13, POLA1, ASPH, RRAS2, PITPNA, UBR5, STUB1, KDM1A, CRYBA2, LEF1, MAPK14, ADAMTS19, KIAA1217, EIF2S3, ENDOV, TMEM47, CDH18, RAPGEF1, MAP3K8, DLG4, STOM, OR1J1, CWF19L1, PRLH, VEGFC, BOLA2, ITGB6, KCNK17, CARS2, EXOC8, SUPT3H, TTC5, TEX28, SHANK1, PDK2, LIN7A, PPP2R5C, OOEP, ST3GAL6, IL34, ZSCAN29, TNK2, NPAS4, TBC1D20, CEP83, LUZP4, SIPA1L1, GPRC5A, CDC42, UACA, SETD5, RBX1, COL4A3BP, SEMA7A, TUB, GTF2H4, WDR60, GRHL3, GABRA2, ZSWIM6, MYL1, TNFSF18, PCGF2, CCDC127, PLCD4, RBL2, USP53, E2F5, OR5AC2, U2AF1, SLC4A7, TTC7A, RPL6, ITPR2, PLK4, CCNT2, KEAP1, RFC4, H2AFX, ELMOD2, KAT2A, B3GLCT, CPQ, BUB1, PIEZO1, POLQ, SOAT1, HCN3, SLITRK6, ATL3, EXOSC8, EHBP1, B4GAT1, ATAD3A, TXNIP, TRIP13, CAMK2G, POLR2D, STAG3, PSTPIP2, ISG15, ZMYM3, TGDS, ELOC, PRAMEF2, CLK1, TNFAIP1, WDR31, BCO1, CPA1, FOXP4, WASHC4, GFM2, RPL26, NDUFB10, RGS5, OPLAH, TUBA4A, CLSTN1, EEF2, MOV10, PRPF4, B3GNT8, FDX1L, ATP6V1B2, ARHGAP18, PTPRN2, CYP26C1, DUSP6, GMPPB, PIMREG, HIVEP2, BEND2, NUCB2, B3GNT4, MTA3, ID4, C11orf40, FAM136A, AKAP1, GAL3ST2, ANOS1, POLR2K, SASS6, RREB1, TBC1D30, NME7, TFDP1, MND1, APOBEC1, SLITRK5, NDST2, TNPO3, LARGE1, TSR2, MBNL1, SYK, HNRNPDL, NECTIN3, TDG, TBR1, PLAG1, FOS, MYCBP2, CYP2D7, CCDC65, XPO1, CAVIN4, PAMR1, CCDC33, EPHA6, FCGR2C, RRM2, NGLY1, CST6, TOP2A, CX3CL1, FAR1, PGAP3, ODAPH, SF3B1, TSG101, ATRIP, NAV1, AEBP1, PSMA7, DBF4B, TUBG1, PCDHB16, IL6ST, PPP4R3A, TEC, CDKN2AIP, APCS, PAK5, CHD4, IKBKE, MRPL40, ACAD10, WNK3, DUT, LYL1, ADH1A, C21orf91, ANGPTL6, TUSC1, DYNC1I1, PPP4C, NFATC2, TREH, KMT2A, RIT1, ERF, SLC12A5, ARL3, MLLT10, COX5B, TMEM121, EIF2AK4, THOC6, TMC3, OTOGL, C7orf43, MSRA, XG, SYNPR, NSMCE3, CEP126, AZIN2, IMPG1, ZNF517, UBQLN1, PPP2R5D, EXTL1, XKR6, WDR5, ATP13A5, FAAP100, RNF219, ST6GALNAC2, ACMSD, DTHD1, CIZ1, WWTR1, RNF212, CBY1, TLE6, SLC35A3, ABRAXAS1, SYNJ1, PCDHA1, SNX1, JAK1, PARP3, ZFR, GALNT11, MFSD4B, ACSL6, CHAF1B, NDUFA3, FLOT1, ULK2, NRCAM, KCNB1, DESI2, SHARPIN, RNF125, rs11130795, CUEDC1, STARD9, GPRIN3, PCSK5, POLR2C, CUX1, ZNF77, TBCK, MARS, RARS, PIGW, SAE1, RSC1A1, ZFP69, FOXD1, IL11, RLIM, FOXD4, ARID4B, GMDS, UBE2V2, BOLA1, ADAM22, CDC5L, APLF, AHNAK2, WNT9B, FAM120B, ADGRV1, MFHAS1, SSBP1, PDIA4, GTF2H3, EOGT, B3GALT1, RBM43, PAM16, DDX3X, KIF3C, SCG5, WAC, USP44, WHRN, PPP4R4, CELF4, SFRP1, CDKAL1, ST8SIA6, VAV3, RMI2, TDP2, STXBP3, DAAM1, MIB1, ADD2, CNTN6, LRIG2, USP1, HCAR1, ZNF420, RBL1, SLC25A43, SMAD5, rs1393978, CDX1, ATRN, DPCD, HAPLN1, NADK2, ARNT2, KCTD1, SPOCK1, SPEF2, DCAF8, ZNF638, CEP250, DLG1, POLR2G, CNTNAP1, KPNA7, APOL2, RNF38, FYB2, AHDC1, GLTP, ITPK1, POLK, GNAL, UBB, TRIM33, PAPOLA, TOP3A, NDUFAB1, COX6C, MS4A10, RPS5, B3GAT1, PUF60, DARS, ELOVL5, EPS8, B4GALNT1, DAD1, LIMS2, SMO, GPC5, MCUB, TMEM240, ZMYND15, CECR2, ANLN, PRR16, PPP3CA, KATNB1, BCLAF1, ALKBH2, FOXJ1, TRA2B, CLEC4E, MGAT4B, RTF1, GTF2H2, FGF17, RBPJ, DAPK3, LMOD3, EMP2, POLE3, PRDM16, TMEM98, FRMD5, ULK4, TRIM2, OTUD4, PPM1K, NFKB2, ELOA2, DENND1A, TMEM99, SLC6A6, ATP1B4, RAB3A, ABRAXAS2, BARX2, NLRP10, NLRP5, TMEM92, EDRF1, RAD18, RANBP6, CRIPT, DEPDC5, RGL1, NKAIN3, FAAP24, ZNF395, RASSF5, ATAD3B, IKZF3, FAM47B, SLC25A21, ARIH1, LGI4, MFAP5, NTAN1, C12orf4, rs37535, MOB4, CLEC3B, NDUFC1, ARL2BP, KIF2A, ZNF79, ARL6IP6, RARB, CCDC18, COG3, MAFA, CBFA2T2, UGT2B4, NAALADL2, UTP14C, FUBP1, CT45A2, TNFSF12, PBX1, ALPP, ASH1L, RDH8, PHACTR1, GPR132, ATP1A1, MTSS1L, PLXNB3, ACVR2A, ANKRD52, MYOD1, SLC39A12, GNAO1, PFKL, TEX13B, AURKB, FAM189B, PRPF39, PROX1, rs6074704, TIPIN, CCDC6, SLC17A9, TOR1AIP1, FOXL1, ADGRG4, BCKDK, CGB3, PFKFB1, POC1A, RAD1, NTM, PHYKPL, GDF2, PCBP3, CHAMP1, TM4SF20, GALNT17, OR13H1, LARGE2, HAGH, VSIG1, CNDP2, ITGB1, BVES, DSTYK, CSPP1, CYB5R4, LRBA, LMAN2L, C1GALT1C1, DSE, FHIT, NFKBIZ, CREB3, ZNF157, GRIA1, SPDL1, FAM81B, ASPSCR1, CCDC90B, MAP1A, EME2, WNT9A, HAS2, CD200, MARCH3, RUNX1T1, DKK4, SMCHD1, RRAS, ZFPL1, BCAS2, FMOD, LUZP2, KCNAB1, BMS1, MXRA5, BPIFB6, NFATC3, POFUT2, GNG2, SELENOS, BCL6, GAL3ST1, MDH2, TXNL4A, RAB25, CRAT, SLC7A11, ZNF780B, VPS53, SPERT, NAA15, BLOC1S5, ASB1, MCTP2, TMEM5, CERS3, PLS3, FIGN, RPL24, PWWP2A, FAM120AOS, ARID2, CALM2, GYG2, PARS2, CTSF, GNA13, B4GALT4, FBXO25, PDK4, GFRA2, NNMT, POLD4, EPHB1, DLL4, UBE2T, POGLUT1, SMARCA1, RECQL5, SSR4, NR2F2, FHOD3, PRDM1, TNRC6B, RERE, PTDSS1, CACNA2D2, FKBP8, POT1, KIAA1109, COX6A1, B3GALT2, CCT7, EEF1A2, rs9388856, HACD1, HERC1, FAM111A, SLA2, HOXB1, INPP5K, CASQ1, INPPL1, BUB3, VIL1, CCDC107, CDC27, CNN2, WNT1, DBP, ZC4H2, KDM5B, ME2, H3F3A, rs12682834, NECTIN1, CGNL1, CLEC4D, ZNF44, EFNA5, NXN, ADA2, IGF2BP2, CENPE, ZIC5, CYP3A7-CYP3A51P, CNTLN, METTL14, SASH1, IL17RD, PHC1, PRAG1, ELOA, NGEF, ZNF582, STK36, MAPK1, HNRNPA2B1, RNF216, GADD45A, SBF1, POSTN, RCHY1, PPP3R1, TACC3, UGGT2, SEM1, DMXL2, STIM2, UBE3B, IQCE, PPP2R5B, SPOP, OSGIN1, LINS1, IKBKB, TCL1A, ENOX1, DOK1, B4GALNT3, PGAP1, NECAP1, POLR2F, ST6GALNAC4, DCLRE1A, RIOK2, PDLIM4, CCNQ, CCPG1, DCBLD1, P3H2, VPS37A, CFL1, OR10V1, PIGQ, NISCH, TENM3, LINGO2, ZNF408, AMBN, CEMIP, SLC35A2, CD74, CTPS1, SYNCRIP, ESS2, KCNJ12, ATP5J, NLRP11, CHRNB3, PID1, TAAR2, RFC1, TRNT1, KIF1BP, MAP2K7, ANKRD44, KIF27, TMEM38B, SMPD3, NFE2L1, GRID2, ZNF423, CHST7, TBX18, RGL2, UTP6, SUZ12, EXTL2, MACC1, CEP19, ELOA3, WWC3, GALNT14, RAB34, TRIM28, POLE4, FGF5, IFNL4, GUCY2F, ERLIN1, CHCHD10, ST3GAL4, DCPS, NFS1, CRMP1, TIMELESS, NUP107, DDX10, MAPRE2, CSNK1G1, RPA3, NLRP4, FGF16, IFT27, DDX59, FLRT3, SEZ6L, NDUFB11, OSBPL2, TBC1D7, VPS54, PRMT9, KANK2, SPP2, ALDH1A3, SPAG1, PPP6C, PRIMPOL, SUCO, GTF2I, KCNN2, BRD2, DGKE, DNAH3, SSPN, SPSB1, ALG5, MESD, DOK2, GALNT8, WDR48, DCAKD, TRAK1, FSBP, EIF4H, DLC1, ACP6, SPRY4, RORC, BOC, MANSC1, STX1B, KPTN, FBXO38, LDB1, HDGFL2, GABBR2, RNF8, SLCO5A1, FCER1G, SNTB1, APEX2, SHOX2, RAD54L2, CPT1C, ARMC5, UBTF, POLD3, TXLNB, POU2F1, COX7C, ERRFI1, MLLT11, ELK3, FAM111B, B4GALT5, TTLL5, MMP19, FBXO28, CLEC10A, INO80, HAP1, B4GALNT2, COL27A1, CCDC78, KDSR, MCM8, OLIG2, LACTB, MID2, CCDC148, ANKS3, PCSK7, HNRNPA1, ZC3H12B, OTOG, ERCC6L2, EPHB4, REEP2, ERC1, VPS45, USP27X, APC2, SLC6A17, STT3B, ZBTB17, B3GNT2, FNBP1, S1PR3, ABHD1, ACTR3C, CDYL, OR52H1, CLPB, ZPLD1, PRTG, TLX3, RCBTB1, SEPT14, FAN1, MED20, DGAT2L6, CTNND2, ANXA1, GUCY1A3, RBFOX3, UTP4, UGGT1, KRTAP9-3, PLCB2, TIA1, HLTF, HMGB3, HMG20B, NUP214, MAGEC3, PRAM1, GPR101, B4GALNT4, RC3H1, MDGA2, RFC3, NSD2, MRI1, CROCC, SMIM3, SLC22A18, MANBAL, PIGZ, RMDN2, CLCN6, CYP2U1, SEPT5, MEOX1, DACH1, CMIP, CARD11, ATP5I, APOL6, PLK1, DAB1, ST3GAL1, DDX47, P3H1, DVL2, DPP4, ZNF141, IKZF1, SLC26A8, HIST2H4A, SNTG1, KCNA2, PCYT1A, XIRP1, THOC2, CYFIP1, COL15A1, CACHD1, EXOC6B, VAMP1, FRRS1L, RIC1, ARNT, MARK1, UBA7, PRPF19, RPA2, REST, CEP170B, MGAT5, IGSF1, FEM1B, TLX2, POLM, ATP5B, POLR2I, NUDT7, rs2988039, NDST4, TSEN15, SDHAF3, CELF6, COL21A1, E2F1, CLP1, CDC25C, CDH2, ACP2, COX7B2, PRAF2, CDX2, LRRC69, TENM2, CAVIN1, MTMR8, TANC1, CACNA1B, SERPINB7, TRH, SLC1A5, ANKS6, C15orf62, CACNG4, ZNF407, SUN1, RPSA, ZP1, STBD1, CXCL8, MGAT5B, DNAJC3, HOXA3, CRY1, PBRM1, RNF20, PIM1, BRF1, FSTL4, PAXIP1, COG2, PNLIP, CDCA7L, MTOR, KCNB2, TENM1, SNRPE, C4orf19, SLC27A1, OAS3, CDK6, P2RY1, RAB27B, SNAP25, KLLN, ZXDA, LRRC29, MYOF, TMTC3, LCK, AFF4, DEFB124, CRB2, UQCRFS1, ST8SIA1, CLDN23, SELENOF, RSPH1, NALCN, KCNE5, SMOX, COPS2, RHBDD2, NCAPG2, PCNA, LMO2, PDPR, B4GALT6, DOLPP1, B4GALT2, SLC38A8, MYCT1, ETV4, CENPS, ALDH1B1, POMK, ARMC4, NICN1, BABAM1, MEGF8, REV1, TRMT44, SALL2, DHTKD1, CRIM1, rs2583136, TULP4, GABARAPL1, IL15, TECPR2, PLEKHA1, LAT2, PKNOX1, NSD3, ABCB5, GALNT7, MAN2A1, SPECC1, VDAC1, TJP1, ZNF599, OR5H6, DEF6, JCAD, LRRTM1, TADA2A, KAT6A, GOPC, SLC35F3, TYRO3, SNX14, CUX2, CCDC151, EXOSC5, CEP89, COA1, CCDC62, ZNF292, ARNTL, RAPGEF4, ZNF813, CTCF, COASY, CCND3, DOT1L, CLASP2, PDE1A, PBX3, TFG, CCDC114, FOXK1, ADGRA2, EMX1, B3GAT2, ANGPT2, THSD7B, DYRK1B, DUOX1, EIF4B, C2CD5, PSMA4, ARSI, STK11IP, ZNF45, ZBTB42, CCNO, ST3GAL2, OGT, CAMKMT, PDGFRL, ASXL3, TREX2, MFAP4, SCAPER, DENND5A, RARA, SS18, TAF6, DLG2, PPP1R12B, DPP3, TUBB2A, DAB2, RBCK1, STARD6, NCKAP5, PLXNA2, MN1, STXBP5L, GPR37, EBF4, OR2J3, CSTF2, DRC1, INHBA, ATP5O, KNL1, DYNAP, DENR, CD80, DDRGK1, KLF8, EPB41L2, PIWIL4, RAP1B, ADAMTS1, PRKG1, RASGRP1, ELAVL4, CDH9, PPM1B, NFIB, MBD6, CAPRIN1, ACKR1, GUCY1A2, FNBP4, SLC35D3, MXI1, SBK3, EXPH5, MAG, SERPINB4, KMT5B, ZNF451, RTTN, NIN, DDIT3, GPR152, GALNTL6, MCM9, CYS1, JUNB, ZKSCAN5, CACNG3, TET2, EPG5, PTH2R, RAB29, ST8SIA3, MDK, PRKD1, MICALCL, CALCRL, SLC22A25, BCL2L1, MAPK3, JARID2, USP34, KIAA1549, CHIC2, MICAL3, SSX1, SLC9A1, GATA5, SORD, ST6GAL2, KIF1C, TMEM231, CABP2, POLN, SEMA3D, AP3D1, PRKACG, SERHL2, KLHL40, FGFR1OP, MINDY2, HSF1, LAS1L, FAT3, POLR2H, PET100, PCDHA3, DSG3, MGAT4A, STK35, DKK1, CCNH, AAGAB, CAPN5, KCNH5, MRPS2, RAD17, INPP5D, ARID4A, ST6GALNAC6, LECT2, SRSF2, TCF3, B4GALT3, SMC5, SLC13A5, BACH1, STAT2, ACTG2, REL, EWSR1, CHRD, LRRFIP2, KLC1, NT5C2, PDK1, IFNA1, MTSS1, CDK5, PADI6, HMMR, CUL2, HEY2, GCG, ARHGDIA, HDHD5, CDK19, AUP1, CETN2, UNC13C, CCND2, CUL4A, PCP4, RIMS2, SYT17, PLD3, SLC6A9, POFUT1, CHSY3, EFCAB13, RTL4, NNT, ALG10, SIK3, SNRPB, ELP1, SS18L1, TIMM21, BCL3, SOX11, C3orf35, DUSP15, CLIP1, ATP5C1, C2orf42, SEMA5A, C1orf127, FAM9C, HEPACAM2, NUMA1, STAC3, CPXCR1, CCDC198, GMPS, GPT2, RIPOR2, TRIT1, CTNNA2, NUDT15, RBFA, ARAF, B3GALNT2, IFNB1, ING1, ETV6, GATAD2B, CRYGA, CCL3, ALKBH3, NANOS1, KDM3A, RPS14, APBB1IP, TBC1D9, LGR4, PGM3, MYH7B, DNAAF4, ZMYM6, ZNF683, ADGRA3, ZNF33A, RNF128, GCNT1, OR51G1, NCOA4, POC1B, FKBP1A, AMD1, PRKCA, TRIB2, COL6A6, TNS2, MAT2A, TUBB4A, RNF6, BOLA2B, WDR93, RUBCN, GRIA2, WDR66, DAZ4, RETREG1, PHF21A, ATP5G1, DNAJC13, USP3, FLRT1, HDAC6, SPART, PLSCR3, XPO5, TOP1, RPS27* |
| TruSight one‡  (not overlapping) | 78 | *GPR98, FAM134B, KIAA1199, KIAA1033, SGK110, WHSC1, PTRF, SPG20, SUV420H1, WDR65, IL8, MFI2, PARK2, GYLTL1B, IKBKAP, HMHA1, PAK7, CGB, KIAA1377, VIMP, GPR56, DGCR14, CIRH1A, KIAA1462, KIAA1432, KIAA1279, KIAA2022, RAB7L1, MURC, DFNB59, LEPRE1, UFD1L, KIAA0196, LINS, GTDC2, DFNB31, DARC, DFNA5, BCMO1, SHFM1, DYX1C1, FAM58A, SEPN1, PYCRL, C10orf11, MESDC2, CYP2D7P1, SETD8, ADC, SEPT15, HSN2, IGKV, PRSS2, PRMT10, C10orf137, KAL1, KIAA0226, DYX1C1-CCPG1, LEPREL1, HEATR2, PVRL4, PVRL1, CYP3A5P1, LARGE, KCNE1L, B3GNT1, B3GALTL, C7orf10, ATP5SL, SGK223, SEPP1, GLTSCR1, CASC5, ADCK3, NAPRT1, SC5DL, PVRL3, ADRBK2* |

* Mitochondrial Nuclear Gene list (mayocliniclabs.com): *AARS2, AASS, ABAT, ABCB7, ACACA, ACAD9, ACO2, AFG3L2, AGK, AIFM1, ALDH3A2, AMPD1, APOPT1, APTX, ATP5A1, ATP5E, ATP5G3, ATPAF2, AUH, BCS1L, BOLA3, C12orf65, CA5A, CHAT, CLPP, COA5, COA6, COQ2, COQ4, COQ6, COQ8A (ADCK3), COQ8B (ADCK4), COQ9, COX10, COX14, COX15, COX20, COX4I2, COX6B1, COX7B, CYC1, D2HGDH, DARS2, DGUOK, DLAT, DLD, DNA2, DNAJC19, DNM1L, EARS2, ELAC2, ETFA, ETFB, ETFDH, ETHE1, FARS2, FASTKD2, FBXL4, FH, FOXRED1, FXN, GAMT, GARS, GCDH, GFER, GFM1, HARS2, HIBCH, IARS2, IBA57, IDH2, ISCU, L2HGDH, LARS2, LIAS, LRPPRC, LYRM4, LYRM7, MARS2, MGME1, MICU1, MPC1, MPV17, MRPL3, MRPL44, MRPS16, MRPS22, MTFMT, MTO1, MTPAP, NDUFA1, NDUFA2, NDUFA9, NDUFA10, NDUFA11, NDUFA12, NDUFAF1, NDUFAF2, NDUFAF3, NDUFAF4, NDUFAF5, NDUFAF6, NDUFB3, NDUFB9, NDUFS1, NDUFS2, NDUFS3, NDUFS4, NDUFS6, NDUFS7, NDUFS8, NDUFV1, NDUFV2, NFU1, NUBPL, OGDH, OPA1, OPA3, OXCT1, PANK2, PC, PCK2, PDHA1, PDHB, PDHX, PDP1, PDSS1, PDSS2, PNKD, PNPT1, POLG, POLG2, PUS1, RARS2, RMND1, RRM2B, SACS, SARS2, SCO1, SCO2, SDHAF1, SERAC1, SFXN4, SLC19A3, SLC25A1, SLC25A3, SLC25A4, SLC25A12, SLC25A19, SLC52A2, SUCLA2, SUCLG1, SUGCT, SURF1, TACO1, TARS2, TAZ, TIMM8A, TIMM44, TK2, TMEM126A, TMEM70, TPK1, TRAP1, TRMU, TSFM, TTC19, TUFM, TWNK (C10orf2), TYMP, UQCRB, UQCRC2, UQCRQ, VARS2, XPNPEP3, YARS2.*

† TruSight expanded: <https://emea.support.illumina.com/content/dam/illumina-support/documents/downloads/productfiles/trusight/trusight-one-expanded-gene-list-v3.zip>

‡ TruSight one: <https://emea.support.illumina.com/content/dam/illumina-support/documents/downloads/productfiles/trusight/trusight-one-gene-list-may-2014.zip>

**Supplemental Table S2. Predictors of Discovering at Least One Variant Classified as “of Uncertain Significance”, “Likely Pathogenic”, or “Pathogenic” in Univariate and Multivariable Analyses.**

|  | Univariate analysis | | | Multivariable analysis† | | |
| --- | --- | --- | --- | --- | --- | --- |
| Predictor | **Beta**  **(SE)** | **Odds ratio**  **(95% CI)** | ***P*-value*** | **Beta**  **(SE)** | **Odds ratio**  **(95% CI)** | ***P*-Value‡** |
| Exome sequencing on the proband and at least one family member | 0.6  (0.24) | 1.83  (1.13–2.95) | 1.16×10^–2^ | 0.60  (0.25) | 1.83  (1.12–2.99) | 0.02 |
| Suspected diagnosis subgroup, Wilson’s disease | -1.73  (0.81) | 0.18  (0.04–0.87) | 1.71×10^–2^ | -1.63  (0.81) | 0.20  (0.04–0.95) | 0.04 |
| Suspected diagnosis subgroup, energy metabolism disorder | -2.56  (1.07) | 0.08  (0.01–0.62) | 1.65×10^–3^ | -2.69  (1.07) | 0.07  (0.01–0.55) | 0.01 |
| Suspected diagnosis subgroup, Ataxia – Hypotonia – Paraparesis | 2.09  (1.05) | 8.06  (1.04–62.61) | 8.45×10^–3^ | Not retained§ | | |
| Suspected diagnosis group, Neurological disorder | 1.1  (0.51) | 3.01  (1.12–8.11) | 1.69×10^–2^ | Not retained§ | | |
| Suspected diagnosis subgroup, Hypercholesterolemia | 0.67  (0.35) | 1.95  (0.98–3.89) | 4.81×10^–2^ | Not retained§ | | |
| Suspected diagnosis group, Liver disorder | -0.63  (0.3) | 0.53  (0.3–0.95) | 3.18×10^–2^ | Not retained§ | | |
| Suspected diagnosis subgroup, Hyperinsulinism | -2.07  (1.1) | 0.13  (0.01–1.08) | 2.50×10^–2^ | Not retained§ | | |

Note. 95% CI: 95% confidence interval; Beta: beta coefficient; SE: standard error.

* Univariate logistic regression analysis.

**†** Cox & Snell R^2^, 0.05; Nagelkerke R^2^, 0.07; Percent of cases correctly classified, 64%; AUROC, 0.589 (95% CI, 0.541 to 0.636).

‡ Multivariable logistic regression analysis using the stepwise method.

§ Not retained in the multivariate logistic regression model

**SUPPLEMENTAL FIGURES**

**Supplemental Figure 1.** Distribution of genetic variants according to their pathogenicity. Variants #1 to #4 are ordered as reported in the CES medical report.


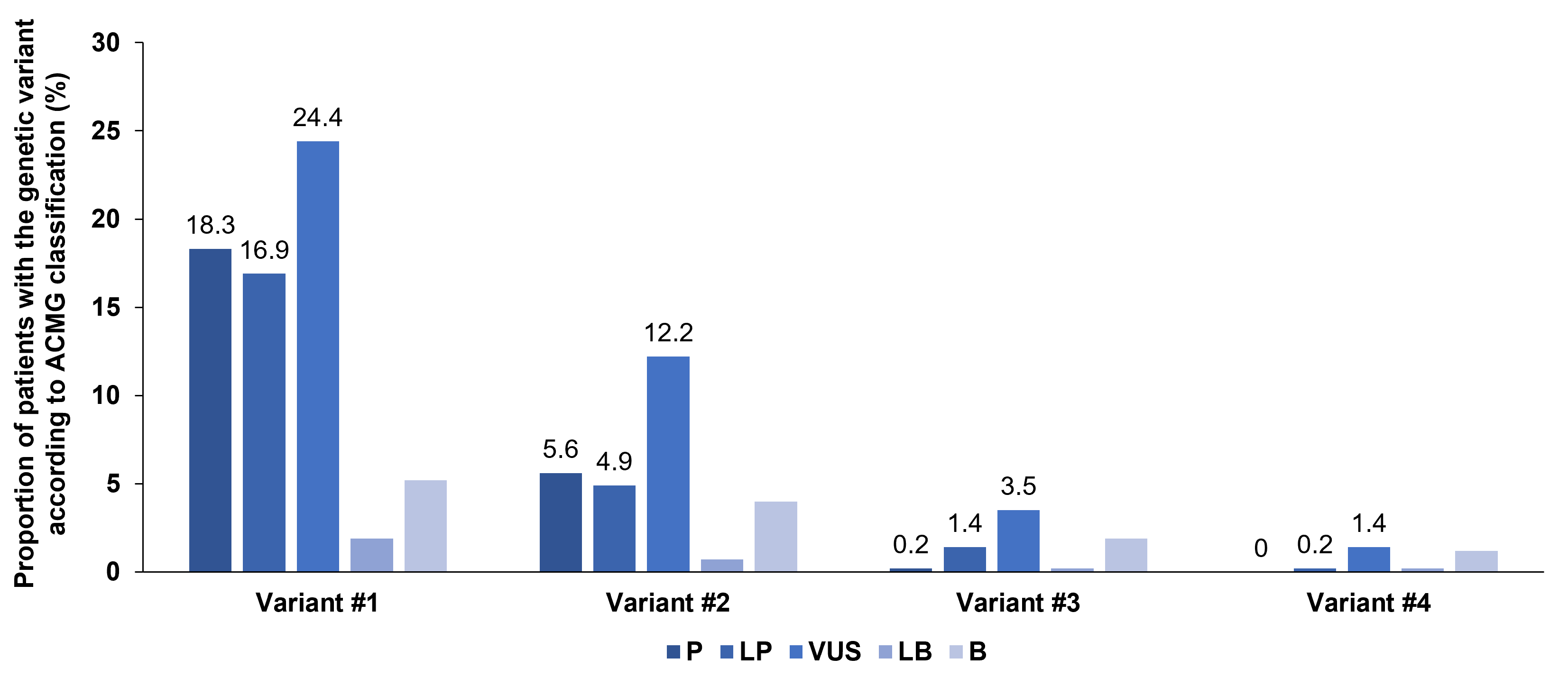


**Supplemental Figure 2.** Distribution of the 203 pathogenic or likely pathogenic variants among the 160 patients with at least one pathogenic (P) or likely pathogenic (LP) variant according to the ACMG criteria. The numbers outside and inside the Venn diagram correspond to the numbers of genetic variants and patients, respectively.


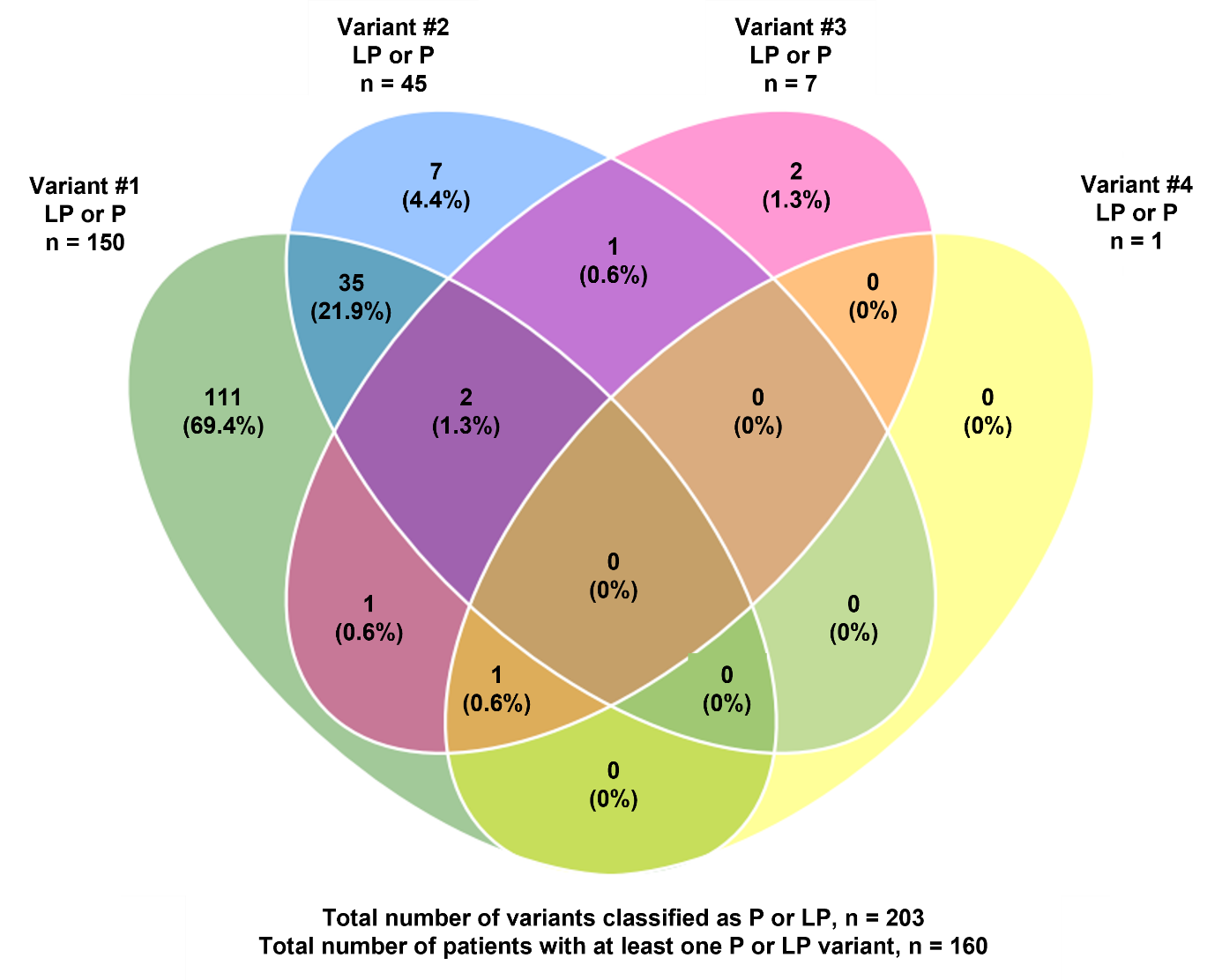


**REFERENCES**

1. Gueant JL, Chery C, Oussalah A, Nadaf J, Coelho D, Josse T, Flayac J, et al. APRDX1 mutant allele causes a MMACHC secondary epimutation in cblC patients. Nat Commun 2018 Jan 4;9 1:67. Epub 20180104 as doi: 10.1038/s41467-017-02306-5.

2. Renard E, Chéry C, Oussalah A, Josse T, Perrin P, Tramoy D, Voirin J, et al. Exome sequencing of cases with neural tube defects identifies candidate genes involved in one-carbon/vitamin B12 metabolisms and Sonic Hedgehog pathway [eng]. Hum Genet 2019 Jul;138 7:703-13. Epub 20190528 as doi: 10.1007/s00439-019-02015-7.

3. Wiedemann A, Chery C, Coelho D, Flayac J, Gueguen N, Desquiret-Dumas V, Feillet F, et al. Mutations in MTHFR and POLG impaired activity of the mitochondrial respiratory chain in 46-year-old twins with spastic paraparesis [eng]. Journal of human genetics 2020 Jan;65 2:91-8. Epub 20191023 as doi: 10.1038/s10038-019-0689-y.

4. Mergnac JP, Wiedemann A, Chery C, Ravel JM, Namour F, Gueant JL, Feillet F, et al. Diagnostic yield of clinical exome sequencing as a first-tier genetic test for the diagnosis of genetic disorders in pediatric patients: results from a referral center study. Hum Genet 2021 DOI: 101007/s00439-021-02358-0 2021 Sep 8. Epub 2021/09/09 as doi: 10.1007/s00439-021-02358-0.

5. Van der Auwera GA, Carneiro MO, Hartl C, Poplin R, Del Angel G, Levy-Moonshine A, Jordan T, et al. From FastQ data to high confidence variant calls: the Genome Analysis Toolkit best practices pipeline. Curr Protoc Bioinformatics 2013;43:11 0 1- 0 33 as doi: 10.1002/0471250953.bi1110s43.

6. Singleton MV, Guthery SL, Voelkerding KV, Chen K, Kennedy B, Margraf RL, Durtschi J, et al. Phevor combines multiple biomedical ontologies for accurate identification of disease-causing alleles in single individuals and small nuclear families. Am J Hum Genet 2014 Apr 3;94 4:599-610 as doi: 10.1016/j.ajhg.2014.03.010.

7. Schwarz JM, Cooper DN, Schuelke M, Seelow D. MutationTaster2: mutation prediction for the deep-sequencing age. Nat Methods 2014 Apr;11 4:361-2. Epub 2014/04/01 as doi: 10.1038/nmeth.2890.

8. Shihab HA, Gough J, Cooper DN, Stenson PD, Barker GL, Edwards KJ, Day IN, et al. Predicting the functional, molecular, and phenotypic consequences of amino acid substitutions using hidden Markov models. Hum Mutat 2013 Jan;34 1:57-65. Epub 2012/10/04 as doi: 10.1002/humu.22225.

9. Dong C, Wei P, Jian X, Gibbs R, Boerwinkle E, Wang K, Liu X. Comparison and integration of deleteriousness prediction methods for nonsynonymous SNVs in whole exome sequencing studies. Hum Mol Genet 2015 Apr 15;24 8:2125-37. Epub 2015/01/02 as doi: 10.1093/hmg/ddu733.

10. Kumar P, Henikoff S, Ng PC. Predicting the effects of coding non-synonymous variants on protein function using the SIFT algorithm. Nat Protoc 2009;4 7:1073-81. Epub 2009/06/30 as doi: 10.1038/nprot.2009.86.

11. Choi Y, Chan AP. PROVEAN web server: a tool to predict the functional effect of amino acid substitutions and indels. Bioinformatics 2015 Aug 15;31 16:2745-7. Epub 2015/04/09 as doi: 10.1093/bioinformatics/btv195.

12. Quang D, Chen Y, Xie X. DANN: a deep learning approach for annotating the pathogenicity of genetic variants. Bioinformatics 2015 Mar 1;31 5:761-3. Epub 2014/10/24 as doi: 10.1093/bioinformatics/btu703.

13. Richards S, Aziz N, Bale S, Bick D, Das S, Gastier-Foster J, Grody WW, et al. Standards and guidelines for the interpretation of sequence variants: a joint consensus recommendation of the American College of Medical Genetics and Genomics and the Association for Molecular Pathology. Genet Med 2015 May;17 5:405-24. Epub 2015/03/06 as doi: 10.1038/gim.2015.30.

14. Landrum MJ, Lee JM, Benson M, Brown GR, Chao C, Chitipiralla S, Gu B, et al. ClinVar: improving access to variant interpretations and supporting evidence. Nucleic Acids Res 2018 Jan 4;46 D1:D1062-D7 as doi: 10.1093/nar/gkx1153.
